# Archaeal Diversity and CO_2_ Fixers in Carbonate-/Siliciclastic-Rock Groundwater Ecosystems

**DOI:** 10.1155/2017/2136287

**Published:** 2017-06-13

**Authors:** Cassandre Sara Lazar, Wenke Stoll, Robert Lehmann, Martina Herrmann, Valérie F. Schwab, Denise M. Akob, Ali Nawaz, Tesfaye Wubet, François Buscot, Kai-Uwe Totsche, Kirsten Küsel

**Affiliations:** ^1^Aquatic Geomicrobiology, Institute of Ecology, Friedrich Schiller University Jena, Jena, Germany; ^2^Department of Hydrogeology, Institute of Geosciences, Friedrich Schiller University Jena, Jena, Germany; ^3^German Centre for Integrative Biodiversity Research (iDiv) Halle-Jena-Leipzig, Leipzig, Germany; ^4^Institute of Geosciences, Friedrich Schiller University Jena, Jena, Germany; ^5^U.S. Geological Survey National Research Program, Reston, VA, USA; ^6^Department of Soil Ecology, Helmholtz Centre for Environmental Research-UFZ, Halle (Saale), Germany

## Abstract

Groundwater environments provide habitats for diverse microbial communities, and although Archaea usually represent a minor fraction of communities, they are involved in key biogeochemical cycles. We analysed the archaeal diversity within a mixed carbonate-rock/siliciclastic-rock aquifer system, vertically from surface soils to subsurface groundwater including aquifer and aquitard rocks. Archaeal diversity was also characterized along a monitoring well transect that spanned surface land uses from forest/woodland to grassland and cropland. Sequencing of 16S rRNA genes showed that only a few surface soil-inhabiting Archaea were present in the groundwater suggesting a restricted input from the surface. Dominant groups in the groundwater belonged to the marine group I (MG-I) Thaumarchaeota and the Woesearchaeota. Most of the groups detected in the aquitard and aquifer rock samples belonged to either cultured or predicted lithoautotrophs (e.g., Thaumarchaeota or Hadesarchaea). Furthermore, to target autotrophs, a series of ^13^CO_2_ stable isotope-probing experiments were conducted using filter pieces obtained after filtration of 10,000 L of groundwater to concentrate cells. These incubations identified the SAGMCG Thaumarchaeota and Bathyarchaeota as groundwater autotrophs. Overall, the results suggest that the majority of Archaea on rocks are fixing CO_2_, while archaeal autotrophy seems to be limited in the groundwater.

## 1. Introduction

Groundwater ecosystems are known to harbor a large range of prokaryotic diversity [1]. In the subsurface in the absence of light, lithoautotrophic prokaryotes can use inorganic compounds as energy sources, thus producing organic compounds available for heterotrophic prokaryotes. Another strategy for prokaryotes to scavenge for carbon and nutrient sources is to attach to aquifer rock surfaces or particles. However, little is known about the microbial diversity attached to percolated solid surfaces (fractures, pores) or within aquifer rock matrices compared to groundwater [2, 3]. In this study, we assessed archaeal diversity in aquifer and aquitard rocks of a mixed carbonate-/siliciclastic-rock aquifer system, as well as in surface soils, along a transect of monitoring wells in the Hainich Critical Zone Exploratory (CZE) in central Germany. Protists (flagellates, ciliates, and amoeba), fungi, bacteria, and Archaea were previously detected via diversity analyses of Hainich CZE groundwater [4]. Quantitative PCR using 16S rRNA genes indicated that on average, Archaea represented 0.03 to 8.2% of the total microbial population across all sites in the Hainich CZE transect [4]. Archaea typically compose the minority fraction of the microbial community in aquifer habitats. Nonetheless, Archaea have been shown to play an important role in key biogeochemical cycles in marine sediments [5] and terrestrial habitats [6]. Since most of the archaeal community diversity in these habitats has been identified using culture-independent 16S rRNA gene surveys, numerous clades of uncultured Archaea have been described, which are diverse and widespread [7]. Until the recent development of metagenomics, little was known about the metabolism of these uncultured Archaea. New archaeal candidate phyla are being described on a regular basis [7–10]; therefore, our knowledge on the physiology and participation of these uncultured Archaea in their environments is constantly growing. A large majority of the Archaea described in terrestrial aquifers are related to either anaerobic methane oxidizers or methanogenic Archaea [11, 12]. However, other studies have reported the presence of a high number of uncultured archaeal clades [13–18]. Hence, broader archaeal diversity and metabolisms are expected in aquifer ecosystems.

**Figure 1:**
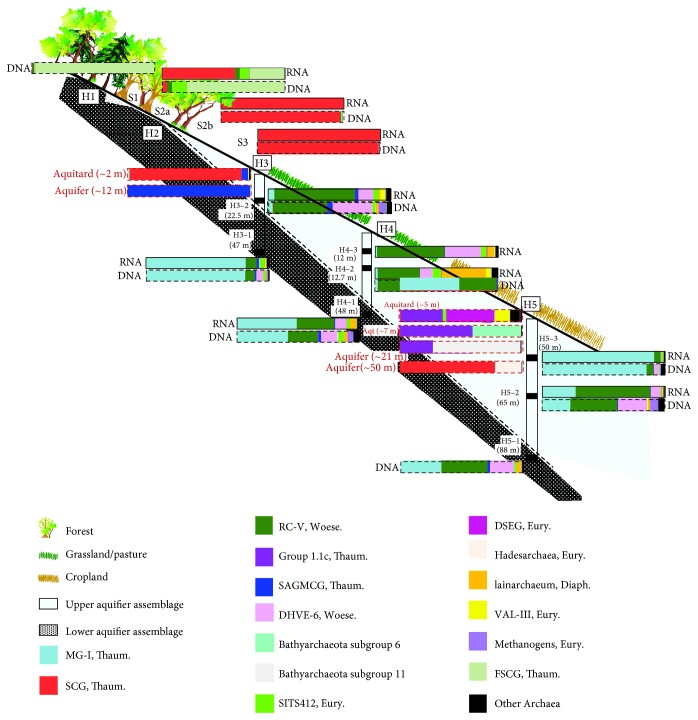
Phylogenetic affiliations of the RNA- and DNA-based archaeal 16S rRNA gene reads in percent of the total reads, in all groundwater samples for August 2014, in aquifer rock samples, and in surface soil samples. Red-dashed boxes represent DNA-based rock samples. MG-I, marine group I; SCG, soil crenarchaeotal group; RC-V, rice cluster V; SAGMCG, South African gold mine crenarchaeotal group; DHVE-6, deep sea hydrothermal vent euryarchaeotal group 6; DSEG, deep sea euryarchaeotal group; VAL-III, Valkea Kotinen lake group III; FSCG, forest soil crenarchaeotal group; Thaum., Thaumarchaeota; Woese., Woesearchaeota; Eury., Euryarchaeota; Diaph., Diapherotrites. S1 to S4 are the soil-sampling sites, and H3–1 to H5–3 are the groundwater wells.

The hill slope well transect of the Hainich CZE passes through a variety of surface land use types, extending from forest (site H1) or unmanaged woodland (national park, site H2) in the Hainich low-mountain ridge area to grassland/cropland (site H3) and cropland agricultural (sites H4 and H5) areas [4] (Figure 1). In the dipping Upper Muschelkalk strata (Middle Triassic), two aquifer assemblages are distinguished in alternating sequence of limestones and marlstones, whose recharge areas are located mainly on the Hainich hill slope. Both superimposed and partially disjointed aquifer assemblages are separated by aquitards, sealing marlstones, leading to substantial differences in their groundwater chemistry. The lower aquifer assemblage (HTL) shows intensive karstification and widened fractures in its single aquifer that allow for faster flow and transport and present variable-dissolved oxygen concentrations [4]. The upper aquifer assemblage, in turn, contains several minor aquifer storeys that are characterized by very low to no dissolved oxygen content (HTU). Moreover, nitrate concentrations are on average lower and ammonium concentrations higher in the upper aquifer assemblage compared to the lower aquifer. Their differences in oxygen availability are probably linked to their respective surface recharge zones and aquifer permeability. Indeed, the aquifers within the upper assemblage receive recharge through the thicker soil cover [4], while the lower aquifer receives quick recharge from the Hainich ridge area characterized by thinner soil cover and forest and woodland land use. Dissolved oxygen was also shown to decrease along the transect following the major flow direction (from sites H1 to H5) [4]. In the downhill direction, the Upper Muschelkalk formations are overlain by younger mixed carbonate/siliciclastic rocks from Lower Keuper and Quarternary age.

In this study, we analysed the DNA- and RNA-based archaeal 16S rRNA gene diversity following different dimensions in the Hainich CZE, in order to reveal the archaeal community structure in this aquifer ecosystem. First, we followed a vertical dimension from the surface soils of the aquifer recharge areas to the carbonate-rock groundwater, in order to assess potential surface to subsurface input of Archaea. Then, we studied archaeal diversity across the groundwater transect. Furthermore, in order to target and identify the archaeal autotrophs and heterotrophs in the aquifers, we conducted a series of ^13^CO_2_ and ^13^C-lignin analogue DNA stable isotope-probing (SIP) experiments using either filtered groundwater or groundwater-exposed aquifer rock pieces.

## 2. Material and Methods

### 2.1. Study Site, Sampling, and Physicochemical Characterization of Groundwater, Soil, and Rock Samples

#### 2.1.1. Groundwater Samples

Water samples were obtained in June, August, and September 2014 from 8 groundwater wells at three sites (H3, H4, and H5) during the regular monthly sampling campaigns within the coordinated joint monitoring program of the Collaborative Research Centre AquaDiva (http://www.aquadiva.uni-jena.de). The wells of the Hainich CZE access two superimposed aquifer assemblages (Hainich transect upper aquifer assemblage (HTU) and Hainich transect lower aquifer assemblage (HTL)) in the thin-bedded alternation of fractured to karstified limestones (aquifers) and marlstones (aquitards) of the Upper Muschelkalk subgroup (Germanic Trias, Thuringian Basin) [4]. We sampled 5 wells of the upper aquifer assemblage (HTU) at mid- to footslope positions of the eastern Hainich slope (depth of well screen section below surface (m): H3-2 (15–22.0), H4–2 (8.5–11.5), H4-3 (8.5–12.5), H5–2 (65.0–69.0), and H5–3 (47.0–50.0). One of the three wells in the lower aquifer assemblage (HTL) at the same sites, developed in the Trochitenkalk formation H3–1 (42.7–46.7) was seasonally dry during these sampling time points, so no water from this site could be collected. Prior to sampling, groundwater was pumped using a Grundfos MP1 submersible pump (Grundfos, Denmark) until physico-chemical parameters stabilized (pH, redox potential, dissolved oxygen, temperature, and specific electrical conductivity) and discharge of at least two well volumes. Water temperature, dissolved oxygen concentration, electrical conductivity, pH, and redox potential were measured on-site in a flow-through cell using a multimeter (Multi 3430, WTW GmbH, Germany), equipped with Tetracon 925, FDO 925, Sentix 980, and ORP 900 probes (WTW GmbH, Germany).

Water samples for DNA and RNA extractions were transferred to sterile glass bottles, and five liters of groundwater were filtered for DNA extractions through 0.2 *μ*m polyethersulfone filters (Supor®, Pall Corporation, USA) on the same day and then stored at −80°C until extraction. Two liters of groundwater were filtered for RNA extractions through a polycarbonate filter (Whatman®, United Kingdom) on the same day, and filters were stored at −80°C until extraction.

#### 2.1.2. Aquifer/Aquitard Rock Samples

Rock samples (Supplemental Table S1 available online at https://doi.org/10.1155/2017/2136287) were collected during the 2010 drilling campaign for the construction of monitoring wells of the Hainich CZE, frozen on dry ice upon recovery in the field, and subsequently stored at −80°C until processed. Drilling was conducted with air or local groundwater as the drilling fluid to avoid contamination. Two rock core samples were recovered from site H3: one from a near-surface loess aquitard (low-permeable rock) and one from a limestone joint aquifer (in which water is mainly moving in fractures and joints). Four rock core samples were recovered from site H5: two from shallow siliciclastic- and carbonate-rock aquitards, one from a deeper siliciclastic porous aquifer (in which water is moving in the porous matrix and fractures), and one from a deep limestone joint aquifer.

In order to avoid contamination from airborne microorganisms during the preparation of rock powder, all processing steps were conducted in a hermetically sealed, stainless steel glove box (UNIlab, M. Braun Inertgas-Systeme GmbH, Germany), which was cleaned with ethanol and DNA-ExitusPlus (AppliChem GmbH, Germany) and flushed with gaseous nitrogen. All additional material and tools used were either autoclaved or when too big to fit in an autoclave rinsed or wiped with ethanol and DNA-ExitusPlus. The rock core samples containing the targeted rock matrix for DNA extractions were sawed into slices (ca. 3 cm thick) with a cut-off saw, using a high-speed diamond-cutting wheel (model K-SC, Kupsch Diamantwerkzeuge GmbH, Germany). The outer slices were discarded. The subsample slices were wrapped in autoclaved aluminium foil and stored at −30°C until further usage. Between samples, the glove box was then cleaned to eliminate the rock dust from sawing and sterilized again. A drill press with diamond hollow drill (12 mm) was used to take plugs from the centre of the rock slices, which were not in contact with drilling mud and other sources of contamination. The obtained rock plugs were then ground with a sterile mortar and pestle. An average 40 g of powder was obtained for each rock core sample and stored at −20° C until extraction of DNA.

#### 2.1.3. Soil Samples

Soil samples were collected in August 2014 in the uphill parts of the Hainich CZE described as recharge areas for both aquifer assemblages (Figure 1). Soil cores were collected using a borer with a diameter of 2 cm. The upper 10 cm of the soil core was used for further processing, and the samples were homogenized using a sterile sieve with 4 mm mesh and then with 2 mm of mesh size in order to remove the roots and stones. At each site, the soils were sampled in 5 adjacent areas with each sampling plot measuring 200 × 150 cm, which was further divided into 15 subplots (50 × 40 cm each). For each time point, 3 triplicate samples from randomly selected subplots were collected. The soil samples were stored on dry ice in the field, and subsequently at −80°C until processed.

### 2.2. Nucleic Acid Extractions from Groundwater, Rock, and Soil Samples and cDNA Synthesis

Groundwater RNA was extracted from half of a filter piece using the PowerWater^®^ RNA Isolation Kit (MO BIO Laboratories Inc., Carlsbad, CA, USA), according to the manufacturer's instructions. Soil RNA was extracted using 2 g of soil and the RNA PowerSoil^®^ Total RNA Isolation Kit (MO BIO, CA, USA). All extracts were stored at −80°C until further analysis. The RNA samples were treated with Turbo™ DNase (Applied Biosystems, USA) and purified using the RNeasy^®^ Mini Kit (QIAGEN, Hilden, Germany). The RNA extracts were tested for genomic DNA contamination by carrying out PCR amplification. RNA extracted from the soil samples was reverse-transcribed as described in Lazar et al. [19] with one modification: random primers (Invitrogen, USA) instead of specific primers were used. cDNA was then stored at −20°C. RNA extracted from the groundwater samples was reverse-transcribed as described in Herrmann et al. [20].

DNA was extracted from half of a filter piece using the PowerSoil DNA Isolation Kit (MO BIO, CA, USA). DNA was extracted from the rock cores using 10 g of rock powder and the PowerMax® Soil DNA Isolation Kit (MO BIO Laboratories Inc., Carlsbad, CA, USA) with slight modifications. Because the limestone powder contained high amounts of calcium, a concentrated phosphate buffer (1 M phosphate buffer/15% ethanol) was used in addition to the kit as suggested by Direito et al. [21]. The phosphate buffer was used in the initial step of the DNA extraction along with the provided solution C1. Because extremely low concentrations of DNA were recovered during the extraction, the DNA eluted in 5 mL of solution C6 provided with the kit was reprecipitated with 20 *μ*g of glycogen (Sigma-Aldrich, USA) and 2 volumes of polyethylene glycol ([30% PEG 6000 and 1.6 M of NaCl], Sigma-Aldrich, USA) at room temperature for 2 hours and then washed with 100 *μ*L 70% ethanol. The DNA pellet was then dissolved in DNase-free molecular grade water and stored at −20°C. Two controls were carried out to test for contamination. One was a test using the MO BIO kit, but using water instead of the rock powder. The other was DNA extraction from rock powder of a core that had been put in an oven for 72 hours at 550°C. The results of the DNA extraction from both controls were sequenced and yielded results for bacterial 16S rRNA gene reads, but none were detected for the archaeal 16S rRNA genes.

### 2.3. ^13^CO_2_-Labelling Experiment Using Rock Pieces Colonized by Groundwater Microorganisms

#### 2.3.1. Sampling

As part of a larger study, passive samplers were designed and constructed to study surface-attached microbial populations in the aquifers. Each passive sampler was packed with autoclaved crushed (1–10 mm diameter) aquifer rock material, deployed in February 2012 in wells H4–1, and retrieved in July 2012. Crushed rocks were collected aseptically from the passive sampler, frozen immediately in the field on dry ice either for characterization of the initial microbial community or for microcosm preparation, and stored on ice in the field then at 4°C in the lab.

#### 2.3.2. Microcosms

Microcosms were constructed in July 2012 by combining 20 g passive sampler material and 40 mL of filtered groundwater from H4–1 in 0.7 L infusion bottles then capped with rubber stoppers and aluminum aperture caps. The headspace was flushed with 100% sterile N_2_ then amended with 10% sterile O_2_ to create an oxic, CO_2_-free system. A total of 12 microcosms were prepared, 9 were amended with ^13^CO_2_ and 3 with ^12^CO_2_. ^13^C-microcosms were amended with ^13^C-bicarbonate (99 atom % ^13^C, Sigma-Aldrich, USA) to a final concentration of 10 mM from a sterile, anoxic stock solution. The headspace was amended with 5% ^13^C-CO_2_ (99 atom % ^13^C, Sigma-Aldrich, USA) to adjust the pH to 7.2. ^12^C-microcosms were amended in the same way with ^12^C-bicarbonate and CO_2_. Microcosms were incubated at 15°C in the dark. After ca. 2 weeks of incubation and no observed oxygen depletion, microcosms were amended with 20 *μ*M final concentration NH_4_Cl. Microcosms were reamended with ^13^C- or ^12^C-bicarbonate every 3 months. After 12 months incubation at 15°C, two ^13^C and one ^12^C-microcosms were sacrificed and the material was collected into sterile 50 mL conical tubes and centrifuged. The pellet and rock pieces were frozen at −80°C until further analysis.

### 2.4. ^13^CO_2_ and ^13^C-Veratric Acid-Labelling Experiment Using Filtered Groundwater from Sites H4 and H5

#### 2.4.1. Sampling

Groundwater samples were obtained from wells H4–1 (oxic aquifer) and H4-3 (anoxic aquifers) in February 2015 and from wells H5–1 (oxic aquifer) and H5–2 (anoxic aquifer) in August 2015. In total ca. 10,000 L groundwater was pumped and filtered on-site from each aquifer, using a submersible pump (Grundfos SQE 5-70, Grundfos, Denmark) connected to a stainless steel filter device (293 mm, Millipore Corp., USA) equipped with a removable precombusted (5 h at 500°C) glass fiber filter (Sterlitech Corp., USA) with a 0.3 *μ*m pore size.

#### 2.4.2. Microcosms

For all samples, 1 L of filtered groundwater was collected and put in 2 L borosilicate glass bottles for use as a medium, to which 500 mg of either ^13^C-labelled sodium bicarbonate (99 atom % ^13^C, Sigma-Aldrich, USA) or unlabelled (^12^C) sodium bicarbonate (Sigma-Aldrich, USA) was added. In addition, only for the groundwater filtered from wells H4–1, 3 bottles were prepared with 70 mg of either ^13^C-labelled veratric acid (Sigma-Aldrich, USA) or unlabelled (^12^C) veratric acid (Sigma-Aldrich, USA). Bottles were sealed with preautoclaved 23.7 mm rubber stoppers with folding skirts (VWR, Germany). After filtration, the filter was cut evenly on a sterile plate and each filter piece was put in the prepared bottles. Before adding the filter piece in the bottles, 120 mL of ^12^C-CO_2_ was added to the headspace of each bottle containing added sodium carbonate. This was done in order to decrease the pH to neutral values (in situ measurements indicated values of ca. 7.2–7.35 in both aquifers). After 5 hours, the pH was measured and was found to be ca. 7.2. For bottles prepared for samples from the anoxic aquifer, the 1 L groundwater with the labelled and/or unlabelled substrates was transferred to 1 L borosilicate glass bottles, which were sealed with rubber stoppers with folding skirts, and flushed with argon for 30 minutes. After adding the filter pieces to the anoxic bottles, the headspace was reflushed with argon for 5 minutes. All bottles were covered in aluminum foil to keep them dark, transported in coolers with ice packs (in situ temperature was on average 10°C), and upon return to the laboratory, the bottles were stored on an agitator (60 rpm) at 15°C for 3 months. pH and oxygen concentrations in the headspace were monitored daily for the first 2 weeks, and subsequently once a week (data not shown). One filter piece from each well was immediately put in dry ice in the field and subsequently stored at −20°C for characterization of the initial microbial community. In order to track potential changes in the microbial community, one third of a filter piece was taken from the ^13^CO_2_ incubation samples using filtered water from wells H4–1 and H4-3, after 4 and 8 weeks (Supplemental Table S2), and filter pieces were stored at −20°C. After 12 weeks incubation, the groundwater medium was filtered using a 0.3 *μ*m glass fiber filter and the remaining filter pieces were stored at −20°C. Oxygen concentrations were measured in the headspace of the oxic incubations, and ammonium, nitrate, sulfate, dissolved inorganic carbon (DIC), and dissolved organic carbon (DOC) were measured in all incubations (beginning and end).

#### 2.4.3. Oxygen Measurements

The oxygen concentration in the gas phase of the oxic incubations was determined by gas chromatography using a 5890A Gas Chromatograph (Hewlett-Packard, USA) with a thermal conductivity detector. The oven temperature was 30°C, the injector temperature was 150°C, and the detector temperature was 175°C. The flow rate was 30 mL/min (20 psi), and we used a molecular sieve 13X, 3 m × 0.125-packed column. Argon was used as the carrier gas.

#### 2.4.4. Ion, DOC, and DIC Measurements

The concentration of NH_4_^+^, NO_3_^−^, and SO_4_^2−^ ions was analyzed using an ion chromatography system (Dionex-DX 500; Thermo Fisher Scientific Inc., USA) at the Max Planck Institute for Biogeochemistry. The samples were injected in a loop of 50 *μ*L or 10 *μ*L depending on the targeted ion concentration. The separation was achieved using an IonPac AS14 analytical column with 3.5 mM Na_2_CO_3_/1.0 mM NaHCO_3_ as the mobile phase at 1.2 mL/min. Detection was carried out with a UV detector and a conductivity detector. The working range was 0.1 to 200 mg/L, and the limit of detection was 0.05 mg/L. The concentrations of DOC and DIC (filter 0.3 *μ*m) were determined by high-temperature catalytic oxidation (multi 18 N/C 2100S, Analytik Jena, Germany) according to DIN EN 1484 [22]. The working range was 0.2 to 100 mg/L.

### 2.5. DNA-SIP Analysis

#### 2.5.1. DNA Extractions

For the SIP incubations using passive sampler material, DNA was extracted from the surface of the rock pieces (6 g per sample) and the centrifuged microcosm pellet, using a protocol based on Barton et al. [23] with modifications. Prior to the addition of proteinase K and sodium dodecyl sulfate (SDS), the samples were mixed with AE buffer and were frozen in liquid nitrogen and then thawed at 65°C three times. In addition to proteinase K, lysozyme (final concentration of 2 mg/mL) was added and the samples were incubated at 37°C for 30 min. Barton et al. [23] use polydexoyinosinic-deoxycytidylic acid (poly-dIdC) as a DNA carrier. However, as it did not increase DNA extraction efficiency and yields in our study, poly-dIdC was not used. For the SIP incubations using filtered groundwater, DNA was extracted from the filter pieces (initial glass fiber filter pooled with the filtered medium after the incubations were stopped) using the RNA PowerSoil Total Isolation Kit followed by the RNA PowerSoil DNA Elution Accessory Kit (MO BIO Laboratories Inc., Carlsbad, CA, USA).

#### 2.5.2. Ultracentrifugation and Fractionation

DNA extracts were separated by CsCl density gradient centrifugation as previously described [24], using an NVT 90 rotor in a XL-70 ultracentrifuge (Beckman Coulter, Krefeld, Germany). After centrifugation, 11 to 12 fractions of 400 *μ*L were collected. DNA from each fraction was precipitated as described in Neufeld et al. [24]. DNA concentrations in each fraction were measured using PicoGreen^®^ (Invitrogen, CA, USA) and a fluorescence microplate reader (Synergy H4, BioTek, VT, USA) (Supplemental Figure S1). Based on the DNA concentrations measured in each fraction and on archaeal 16S rRNA gene-denaturing gradient gel electrophoresis gel profiles used for screening the different fractions (data not shown), specific DNA fractions were pooled and/or chosen as representative of the light (density < 1.715 g/mL) and heavy fractions (density ≥ 1.72 g/mL) for each sample (Supplemental Figure S1) and subsequently used for illumina sequencing.

### 2.6. Illumina Sequencing

Samples were shipped to LGC Genomics GmbH (Berlin, Germany) for illumina MiSeq sequencing with the PARCH340F-Arch915R [25, 26] primer pair. This primer pair is commonly used to amplify 16S rRNA genes in marine environments because it specifically targets the archaeal domain and was estimated to cover 66% of archaeal phylotypes in marine subsurface habitats [5]. However, reverse primer A915R was shown to have mismatches with three archaeal groups, and thus archaeal diversity is probably underestimated with this primer pair. Because the DNA concentrations from the aquifer rock extracts were extremely low, PCR products were used for sequencing, and not genomic DNA. This initial round of PCR was carried out on the aquifer rock DNA samples using the A24F-A1492R [27, 28] primer pair, and conditions for PCR were 35 cycles with 1 min at 94°C, 1 min at 49°C, and 2 min at 72°C. The illumina sequence datasets were analyzed together using mothur v.1.36.1 [29], following the Schloss SOP (http://www.mothur.org/wiki/MiSeq_SOP). Pair ends obtained after sequencing were merged in mothur, and after processing, the sequence reads were 575 bp. Archaeal taxonomy was initially assigned using the Silva reference database [30], then manually checked, and reassigned for some groups, using a personal reference database in ARB [31] containing a higher diversity of uncultured archaeal taxa and newly described archaeal candidate phyla. Sequences obtained in this study were deposited in the European Nucleotide Archive under Project no. PRJEB14009, accession numbers ERR1414291-ERR1414336 for the groundwater samples, ERR1414343-ERR1414386 and ERS1403683-ERS1403696 for the SIP incubations samples, ERR1414387-ERR1414399 for the rock samples, and ERR1414400-ERR1414422 for the soil samples.

### 2.7. Statistical Analyses

#### 2.7.1. Cluster Dendrogram

A cluster dendrogram was computed using R [32] with the pvclust package [33]. The dendrogram was calculated using the correlation distance and the Ward agglomeration methods.

#### 2.7.2. DNA-SIP Analysis

To estimate the enrichment of a specific archaeal taxon in the heavy DNA fractions compared to the light fractions of the ^13^C and ^12^C SIP incubations, we used the odds ratio (OR) following the formula:

(1) $$\begin{array}{c} OR=\frac{\left(H_{arc}/H_{narc}\right)}{\left(L_{arc}/L_{narc}\right)}, \end{array}$$

where *H*_arc_ is the number of reads for a specific archaeal taxon in the heavy fraction, *H*_narc_ the total number of reads in the heavy fraction minus *H*_arc_, *L*_arc_ the number of reads for the specific archaeal taxon in the light fraction, and *L*_narc_ the total number of reads in the light fraction minus *L*_arc_. Only incubations with ≥5 reads in both the heavy and light fractions were used for analysis. An odds ratio > 1 for a given archaeal taxon suggests that it is more abundant in the heavy fraction than in the light fraction. Then the ratio of the odds ratios (RoOR) between ^13^C and ^12^C incubations was calculated for both ^13^C duplicate samples. A RoOR > 1 for a given archaeal taxon indicates that the taxon is more enriched in the heavy fraction of the ^13^C incubation compared to the control ^12^C incubation, suggesting that the archaeal taxon was labelled.

## 3. Results and Discussion

### 3.1. Archaeal Diversity from Surface Soils to the Subsurface

**Figure 2:**
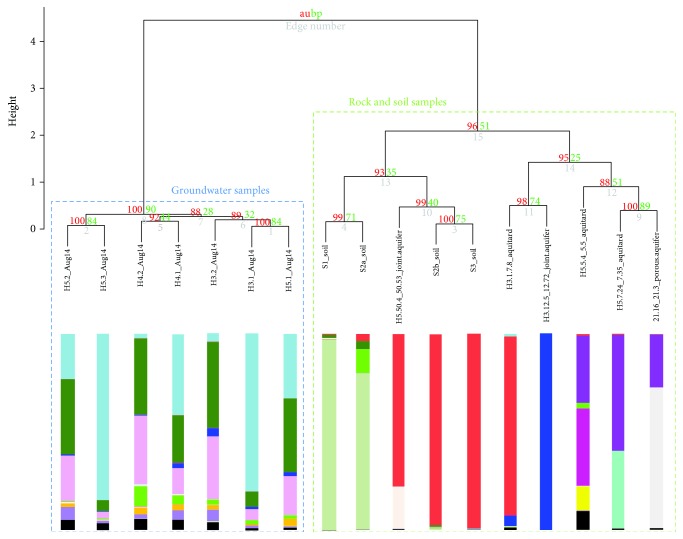
Dendrogram from cluster analysis of DNA-based archaeal 16S rRNA gene diversity, using the R software and pvclust package. Bar indicates dissimilarity values. Approximately unbiased (AU) *p* values are shown in red, and bootstrap probability (BP) values are shown in green at each node. Histograms represent the phylogenetic affiliations of the DNA-based archaeal 16S rRNA gene reads shown in Figure 1.

Archaeal diversity was assessed from surface soil samples in the recharge areas of both aquifers to the aquifer/aquitard rocks and groundwater, in order to gain a better understanding of subsurface archaeal transport in the Hainich CZE. Clustering of groundwater, aquifer rock, and soil samples based on archaeal 16S rRNA genes showed that the archaeal diversity in these three habitats were distinct, with a clear separation of the groundwater compared to the soil and rock samples (Figure 2), indicating the existence of a unique archaeal population in the groundwater of both aquifer assemblages. The DNA- and RNA-based archaeal community in the two soil samples from the recharge area of the upper aquifer (S2b and S3) was almost solely composed of the soil crenarchaeotal group (SCG or group 1.1b, whose cultured representatives belong to the *Nitrososphaerales* order) Thaumarchaeota clade (Figure 1, Supplemental Figure S2, and Table S3), which made up on average 0.03% of the groundwater Archaea in the upper aquifer assemblage, and also in minor proportion the SITS412 Thermoplasmatales and Woesearchaeota. Furthermore, the near-surface loess aquitard rock sample (Figure 1, Supplemental Table S1) recovered from site H3 was dominated by the SCG Thaumarchaeota, whereas the rock sample from the deeper joint aquifer was almost solely composed of the South African gold mine miscellaneous crenarchaeotal group (SAGMCG, whose cultured representatives belong to the *Nitrosotaleales* order) Thaumarchaeota. The porous aquifer sample from site H5 was dominated by Bathyarchaeota subgroup 11, whereas the deeper limestone aquifer sample was composed of SCG Thaumarchaeota as well as Hadesarchaea (Figure 1, Supplemental Table S3). Thus, these observations suggest seepage of Archaea, such as the SCG Thaumarchaeota, from the soils to the shallow aquitard at site H3, and to the groundwater at sites H3 and H4. This is supported by a previous study suggesting active transport of fungi by water flow in the subsurface of the Hainich CZE [34]. Moreover, the relative abundance of SITS412 Thermoplasmatales and Woesearchaeota increased in the anoxic groundwater samples, suggesting that these Archaea thrive better in the subsurface groundwater than in the surface soils.

Conversely, the forest soil crenarchaeotal group (FSCG or group 1.1c) and the SCG Thaumarchaeota, as well as the SITS412 Thermoplasmatales were the dominant metabolically active Archaea, as indicated by RNA-based sequencing, in the surface forest and woodland recharge locations (S1 and S2a) of the lower oxic aquifer assemblage (Figure 1, Supplemental Table S3). However, neither of these archaeal groups was detected in the oxic groundwater samples from sites H3 to H5, implying that the soil Archaea transported from the forest and woodland locations cannot survive in the groundwater habitat. Nonetheless, since wells from forest sites H1 and H2 did not contain sufficient amounts of water for sampling, filtration, and diversity analyses, it is possible that the FSCG and SCG Thaumarchaeota are found in the groundwater closest to the recharge areas.

### 3.2. Archaeal Diversity in Groundwater

Archaeal diversity was assessed in groundwater from all eight wells at three different time points (June, August, and September 2014), using RNA and DNA (Supplemental Figure S3). This analysis showed mostly consistent results suggesting reproducibility of results obtained from the different wells over different time points. However, RNA-based archaeal diversity from wells H5–2 sampled in September 2014 and the DNA-based archaeal diversity from wells H3–1 sampled in June and August 2014 differed. For the following discussion, we will focus on the groundwater archaeal diversity from August 2014. In the upper aquifer assemblages, the archaeal community at H3-2 was dominated by the rice cluster V (RC-V) (Figure 1, Supplemental Figure S2, and Table S3) and the deep sea hydrothermal vent euryarchaeota group 6 (DHVE-6) both belonging to the Woesearchaeota candidate phylum. The marine group I (MG-I or group 1.1a, whose cultured representatives belong to the *Nitrosopumilales* order) Thaumarchaeota were the third most abundant detected group.

A similar distribution of RC-V, DHVE-6, and MG-I was observed at H4–2, H4-3, and H5–2. At H5–3, both DNA- and RNA-based archaeal communities showed a dominance of MG-I over RC-V and DHVE-6. In wells H4–2 and H4-3, a relative increase of the Iainarchaeum belonging to the Diapherotrites [35] and of the SITS412 clade belonging to the Thermoplasmatales order within the Euryarchaeota phylum could also be observed. Both the RNA- and DNA-based archaeal communities in the lower oxic aquifer assemblage were dominated by the MG-I Thaumarchaeota and the RC-V and DHVE-6 Woesearchaeota (Figure 1, Supplemental Table S3). The relative abundance of RNA- and DNA-derived reads affiliated with the MG-I decreased from wells H3–1 to wells H4–1 and H5–1, and the RC-V and DHVE-6 Woesearchaeota increased from wells H3–1 to wells H4–1 and H5–1. This could be explained by the decreasing oxygen concentrations along the transect (Supplemental Table S4). The RC-V and DHVE-6 Woesearchaeota, both uncultured archaeal groups, represented a major part of the active archaeal community in both aquifer assemblages (except at wells H3–1 and H5–3). Genomic reconstruction of these Archaea predict that the RC-V are strict fermenters metabolizing plant-derived carbohydrates [36] and that the DHVE-6 mainly have a symbiotic or parasitic lifestyle [7]. In both aquifer assemblages, the MG-I Thaumarchaeota was the other major group that was metabolically active in the groundwater. One cultured representative was shown to be an aerobic ammonia-oxidizing archaeon [37], supporting a previous study using functional genes indicating the presence of archaeal ammonia oxidizers in the Hainich CZE groundwater transect [38]. The groundwater samples from all wells contained various concentrations of NH_4_^+^ which could thus be available for oxidation (Supplemental Table S4).

### 3.3. Stable Isotope-Probing Incubations with Groundwater

**Figure 3:**
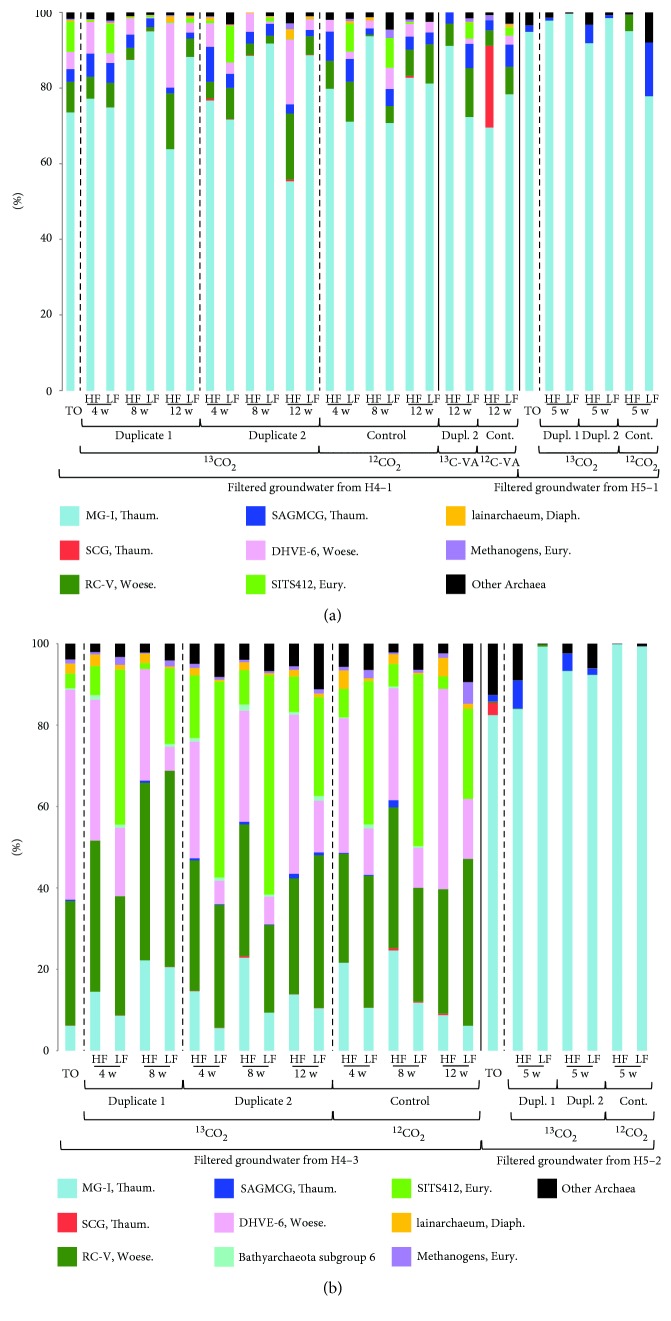
(a) Phylogenetic affiliations of archaeal 16S rRNA gene reads as a percent of total reads in the initial filter pieces used before SIP incubation (T0) and in the SIP heavy (HF) and light (LF) DNA fractions from the oxic H4–1 and H5–1 aquifers; (b) phylogenetic affiliations of archaeal 16S rRNA gene reads as a percent of total reads, in the initial filter pieces (T0) and in the SIP heavy (HF) and light (LF) DNA fractions from the anoxic H4-3 and H5–2 aquifers. Dupl., duplicate; w, week; VA, veratric acid.

Analysis of the archaeal 16S rRNA gene diversity in the Hainich CZE aquifer systems unearthed many uncultured clades. Thus, in order to gain insight into potential substrates used by the Archaea, we conducted a series of oxic and anoxic incubations using groundwater from oxic wells H4–1 and H5–1 and anoxic wells H4-3 and H5–2 with labelled ^13^CO_2_; and with labelled ^13^C-veratric acid using groundwater only from oxic wells H4–1. Because the microbial biomass in the groundwater is usually too low for direct use in SIP experiments, we increased the biomass by filtering 10,000 L of groundwater and used these filter pieces for incubation. This high-volume filtration process lasted approximately ten hours, which could have impacted the community structure of the archaeal populations. Comparison of the archaeal diversity in the initial filter piece frozen immediately in the field (T0 in Figures 3(a) and 3(b)) and the archaeal diversity observed during the regular sampling campaigns (Figure 1, Supplemental Figure S3) suggests that the overall archaeal community was not substantially changed for the groundwater from wells H4–1 and H4-3, but an increase of the relative amounts of the MG-I Thaumarchaeota for wells H5–1 and H5–2 was observed. Therefore, the longer filtration time most likely impacted the archaeal community for wells H5–1 and H5–2, resulting in the loss of Woesearchaeota which dominated the groundwater samples of the regular sampling campaigns.

Veratric acid is a lignin-related aromatic compound and a complex organic polymer derived from plants and algae [39]. It was used as substrate to target heterotrophs because it was assumed that the available organic matter in the subsurface would be plant-derived complex carbohydrates. In this study, we used a ratio of odds ratios (RoOR) between the ^13^C duplicate samples and the ^12^C control sample, to estimate labelling for a given archaeal taxon. The MG-I Thaumarchaeota had a RoOR > 1 in one duplicate of the oxic ^13^C-veratric acid-labelled sample from wells H4–1, which indicates that they are more enriched in the heavy fraction in the ^13^C bottles compared to the ^12^C control, suggesting that they were labelled (Figure 3(a), Supplemental Table S5).

Since autotrophy is thought to play an important role in the subsurface, we focused on this group of Archaea by carrying out incubations with labelled ^13^CO_2_. Given the lack of information on SIP using groundwater and ^13^CO_2_, we decided to analyze different time points from the first series of incubations using groundwater from site H4 (wells H4–1 and H4-3), to test for optimal incubation time. Thus, labelling of archaeal taxa was monitored after 4, 8, and 12 weeks incubations. The RC-V Woesearchaeota and the Bathyarchaeota subgroup 6 had a RoOR > 1 in the anoxic ^13^CO_2_-labelled samples from wells H4-3, suggesting that they were labelled (Figure 3(b), Supplemental Table S5), with the caveat that a low number of sequences were detected in the heavy fractions for the Bathyarchaeota subgroup 6. And, the DHVE-6 Woesearchaeota and SAGMCG Thaumarchaeota had a RoOR > 1 in the oxic ^13^CO_2_-labelled samples from wells H4–1 (Figure 3(a), Supplemental Table S5).

Since labelled DNA was observed after 4 weeks incubation during this first series of SIP experiments, we decided to incubate the second series of groundwater samples from site H5 (wells H5–1 and H5–2) for 5 weeks. The SAGMCG Thaumarchaeota had a RoOR > 1 in the oxic incubations using groundwater from wells H5–1, indicating that they were labelled (Figure 3(a); Supplemental Table S5).

Oxygen concentrations were measured in the oxic incubations with ^13^CO_2_ or ^13^C-veratric acid from wells H4–1 and H5–1, at a constant temperature of 15°C, and showed a decrease from ca. 8–8.9 mg/L at the start of the incubations down to 6.8–8 mg/L at the end (Supplemental Table S6), indicating aerobic respiration in the oxic bottles. Nitrate and sulfate concentrations decreased during the anoxic incubations from wells H4-3 and H5–2, indicating that anaerobic respiration also likely occurred using these electron acceptors (Supplemental Table S6). Furthermore, apart from the incubations using groundwater from wells H4-3, all others showed an increase in DOC concentrations, a sign that CO_2_ was fixed during the SIP experiments (Supplemental Table S6).

### 3.4. Autotrophic and Heterotrophic Archaea in the Subsurface

The MG-I were the dominant Archaea in the heavy DNA fractions of the ^13^CO_2_ incubations with filtered groundwater from the anoxic wells H5–2 (Figure 3(b)) and from the oxic wells H4–1 and H5–1 (Figure 3(a)). However, surprisingly, there was no evidence that the MG-I were labelled during these experiments (Supplemental Table S5). But, the MG-I were the only labelled taxon in the incubations with ^13^C-veratric acid using filtered groundwater from wells H4–1. This suggests that the MG-I Thaumarchaeota in the oxic groundwater from the lower aquifer assemblage mainly have a chemoorganotrophic lifestyle, which has already been suggested for planktonic MG-I [40, 41], or that they have a mixotrophic metabolism. Nonetheless, further incubations using water from the other wells would be necessary to confirm this observation. Both Woesearchaeota subgroups RC-V and DHVE-6 were estimated to be labelled in the ^13^CO_2_ incubations using groundwater from sites H4-3 and H4–1. However, the available reconstructed genomes for both these groups predict strict heterotrophic metabolisms or even symbiotic lifestyles [7, 36]. One side effect of longer SIP experiments is cross-feeding, during which a secondary group of organisms takes up ^13^C-labelled metabolites produced by the primary users of the ^13^C–labelled substrate. Therefore, it is possible that these Woesearchaeota were labelled because they used ^13^C-labelled organic compounds produced by the ^13^CO_2_ fixers.

In the subsurface, when the input of surface-derived fresh organic matter is limited, heterotrophic communities are thought to rely on carbon fixers to produce organic carbon [42]. The importance of chemolithoautotrophy in groundwater environments has been highlighted in studies based on the analysis of functional genes specific for CO_2_ fixation pathways, in sites such as polluted aquifers [43, 44] or acetate-amended aquifers [45]. A previous study of lithoautotrophic bacteria in the Hainich transect aquifers indicated that a considerable fraction of the total bacterial population (1–17%) has the potential to fix CO_2_ [46]. In this study, the ^13^CO_2_ SIP experiments suggested that the Bathyarchaeota subgroup 6 fixed CO_2_ in wells H4-3 and that the SAGMCG Thaumarchaeota fixed CO_2_ in wells H4–1 and H5–1. However, because of the long incubation times, we cannot exclude that these groups were labelled due to cross-feeding. Nonetheless, previous reconstruction of different Bathyarchaeota subgroup genomes predict that they are facultative autotrophs using the reductive acetyl-CoA pathway [47]. A cultured representative of the SAGMCG Thaumarchaeota was described as an acidophilic ammonia-oxidizing chemolithotroph [48], using the 3-hydroxypropionate-4-hydroxybutyrate pathway [49]. Moreover, although NH_4_^+^ concentrations were below the detection limit in the oxic incubations, nitrate concentrations increased in the incubations using groundwater from wells H4–1 (Supplemental Table S6), suggesting ongoing ammonia oxidation. Thus, the groundwater from the oxic and anoxic aquifer assemblages seems to harbour distinct archaeal CO_2_ fixers, and these differences are most likely explained by oxygen availability.

Quantitatively, the Calvin-Benson-Bassham cycle, which is mostly used by bacteria, is the most widespread CO_2_ fixation pathway in nature [50]. However, the different carbon fixation pathways used by the Archaea are thought to reflect their geochemical environment. For example, the acetyl-CoA pathway has the lowest energetic cost (less than one ATP molecule can result in the production of pyruvate), and the 3-hydroxypropionate-4-hydroxybutyrate pathway can use bicarbonate [51]. Both the Bathyarchaeota subgroup 6 and the SAGMCG Thaumarchaeota were detected only in small amounts in the initial groundwater filter pieces before incubation (0.35 and 3.4% of the total reads, resp.), suggesting that CO_2_ fixation carried out by these Archaea in the groundwater could be limited. However, it is also possible that there was no energy (such as lack of suitable or available inorganic electron donors) for CO_2_ assimilation by some archaeal groups during the incubations. It is also possible that we did not detect all the autotrophs, especially for the groundwater from site H5, given the shift towards an increase in the relative abundance in MG-I Thaumarchaeota during the high-volume filtration.

In comparison, the dominant archaeal groups that were detected in the upper anoxic aquifer rock samples, that is, the SAGMCG and SCG Thaumarchaeota at site H3 and the Bathyarchaeota subgroups 6 and 11 and the Hadesarchaea [9] at site H5, are (predicted) lithoautotrophs. Indeed, the SCG and SAGMCG Thaumarchaeota were both first detected in gold mine waters in South Africa [18], and a cultivated member of the SCG was described as a chemolithoautotrophic ammonia-oxidizing archaeon [52, 53]. And, based on genome reconstruction, the Hadesarchaea are predicted to be strictly anaerobic, lithoautotroph-oxidizing H_2_ or CO-coupled to anaerobic nitrite reduction to ammonium, and S^0^ reduction to sulfide, which would help them survive in the subsurface biosphere [9]. These archaeal groups were either not detected or detected in extremely low amounts in the groundwater samples from H3-2, H5–2, and H5–3. Because both rock and soil samples were recovered at different time points, direct comparison is not fully relevant, and this could imply that these (predicted) lithoautotrophic Archaea might prefer living in the aquifer rocks.

Since sequencing of the DNA recovered from rock samples of the lower aquifer was either unsuccessful or yielded only a few reads, the archaeal diversity attached on these rock surfaces was studied by exposing autoclaved aquifer rock pieces (passive sampler material) in wells H4–1. After 6 months exposure, the surface of the rocks was mainly colonized by MG-I (53.6% of the total reads) and SCG (38.7%) Thaumarchaeota (Supplemental Figure S4). After retrieving the passive sampler material, we conducted a second series of ^13^CO_2_ SIP incubations using this rock material. The MG-I and SCG Thaumarchaeota on the passive sampler material before incubation were suggested to be labelled after a one-year incubation (RoOR > 1, Supplemental Table S5). The long incubation period of this SIP experiment makes it difficult to distinguish between labelled autotrophic and heterotrophic Archaea due to cross-feeding. Nevertheless, both of these Thaumarchaeota have been described as facultative lithoautotrophs [53, 54]. Therefore, it is possible that the major fraction of the Archaea that attached to the rock surface in the lower aquifer assemblages are CO_2_ fixers, confirming our previous observation.

## 4. Summary and Conclusions

The Archaea detected in soils of the recharge areas of the upper aquifer assemblage were also detected in the groundwater from this assemblage, suggesting input of Archaea from the surface to the aquifer. However, the Archaea detected in soil samples of recharge areas of the lower aquifer assemblage were not detected in the oxic groundwater, possibly indicating these Archaea could not survive the conditions in the subsurface. The DNA- and RNA-based archaeal 16S rRNA gene surveys of the upper anoxic and lower oxic aquifer assemblages indicated that the Thaumarchaeota and the Woesearchaeota dominated the groundwater habitat. The results of the SIP incubations suggested that the MG-I Thaumarchaeota did not fix CO_2_ but used a lignin analogue as energy source in one of the transect wells. The ^13^CO_2_-labelling experiments using filtered groundwater indicated that the estimated labelled archaeal taxa did not constitute a major portion of the archaeal community in the groundwater, hinting that only a small fraction of the Archaea have the potential to fix CO_2_ in groundwater. Furthermore, the Archaea detected in rock samples from anoxic aquifers were distinct from those detected in anoxic groundwater and were dominated by predicted lithoautotrophic Archaea. Overall, this study highlighted a unique archaeal population found within the aquifer habitats of the Hainich CZE. Further work such as archaeal metagenomic surveys should help understand the involvement of these Archaea in carbon cycling in this ecosystem.

## Supplementary Material

Figure S1. Concentrations of DNA from each fraction, for each sample, obtained using PicoGreen staining. DNA concentrations of the control (^12^C) bottle samples are shown in dashes. For more details on the Bottle IDs please refer to the Supplemental Table S2. LF, light fraction; HF, heavy fraction. Figure S2. Phylogenetic trees showing the affiliations of selected representative archaeal 16S rRNA gene reads from groundwater (GW), soil and rock, SIP using filtered groundwater (SIPf or SIP filter), and SIP using passive sampler material (SIPps or SIP passive sampler) samples. The trees were calculated with 575 bp by neighbor-joining analysis in ARB. Bootstrap values are based on 1,000 replicates and are indicated at nodes for values ≥50 %. (A) Thaumarchaeota, (B) Euryarchaeota, (C) Woesearchaeota, and (D) Bathyarchaeota. Figure S3. Dendrogram from cluster analysis of archaeal 16S rRNA gene diversity from the groundwater samples recovered in June, August and September 2014, using the R software and the pvclust package. Bar indicates dissimilarity values. Approximately Unbiased (AU) p-values are shown in red, and Bootstrap Probability (BP) values are shown in green at each node. Histograms represent the phylogenetic affiliations of the RNA-based (A) and DNA- based (B) 16S rRNA gene reads. Color legends are the same as in Figure 1. Figure S4. Phylogenetic affiliations of archaeal 16S rRNA gene reads in percent of total reads, in the colonized passive sampler material exposed in well H4-1 for 6 months (T0), and in the SIP heavy (HF) and light (LF) DNA fractions. y, year. Table S1. List of the rock cores samples used for DNA extraction and 16S rRNA gene diversity analysis. Table S2. List of the different types of material, labelled substrates, incubation times and conditions used for the SIP experiments. Table S3. Relative proportion (%) of archaeal taxa in each soil (August 2014), rock and groundwater (June, August and September 2014) sample, based on 16S rRNA gene diversity. Table S4. Physicochemical parameters measured in groundwater during sampling, from all sampled wells at the 3 time points (June, August and September 2014). Table S5. Phylogenetic affiliation of archaeal 16S rRNA gene reads in relative in the light (LF) and heavy (HF) DNA fractions from the anoxic H4-3 and H5-2 and oxic H4-1 and H5-1 groundwater samples (filter pieces) and H4-1 passive sampler material; using labelled ^13^C-CO2 and ^13^C-labelled veratric acid (VA). Only incubation bottles for which both fractions had ≥5 reads are shown. The odds ratio (OR) and ratio of odds ratio (RoOR) were calculated. VA, Veratric Acid. Table S6. Physicochemical parameters measured in groundwater during the ^13^CO2 and ^13^C-veratric acid SIP incubations using filtered groundwater from the oxic and anoxic aquifers. VA, veratric acid; na, not applicable; nd, not detected; T0, start of the incubation; Te, end of the incubation; DOC, dissolved organic carbon; DIC, dissolved inorganic carbon.
