# Supplementary material for: Archaeal Diversity and CO2 Fixers in Carbonate-/Siliciclastic-Rock Groundwater Ecosystems
Source: Archaea. 2017 Jun 13;2017:2136287. doi: 10.1155/2017/2136287 (PMC5485487; doi:10.1155/2017/2136287)
Supplement: Supplementary file 1 — Figure S1. Concentrations of DNA from each fraction, for each sample, obtained using PicoGreen staining. DNA concentrations of the control (12C) bottle samples are shown in dashes. For more details on the Bottle IDs please refer to the Supplemental Table S2. LF, light fraction; HF, heavy fraction. Figure S2. Phylogenetic trees showing the affiliations of selected representative archaeal 16S rRNA gene reads from groundwater (GW), soil and rock, SIP using filtered groundwater (SIPf or SIP filter), and SIP using passive sampler material (SIPps or SIP passive sampler) samples. The trees were calculated with 575 bp by neighbor-joining analysis in ARB. Bootstrap values are based on 1,000 replicates and are indicated at nodes for values ≥50 %. (A) Thaumarchaeota, (B) Euryarchaeota, (C) Woesearchaeota, and (D) Bathyarchaeota. Figure S3. Dendrogram from cluster analysis of archaeal 16S rRNA gene diversity from the groundwater samples recovered in June, August and September 2014, using the R software and the pvclust package. Bar indicates dissimilarity values. Approximately Unbiased (AU) p-values are shown in red, and Bootstrap Probability (BP) values are shown in green at each node. Histograms represent the phylogenetic affiliations of the RNA-based (A) and DNA- based (B) 16S rRNA gene reads. Color legends are the same as in Figure 1. Figure S4. Phylogenetic affiliations of archaeal 16S rRNA gene reads in percent of total reads, in the colonized passive sampler material exposed in well H4-1 for 6 months (T0), and in the SIP heavy (HF) and light (LF) DNA fractions. y, year. Table S1. List of the rock cores samples used for DNA extraction and 16S rRNA gene diversity analysis. Table S2. List of the different types of material, labelled substrates, incubation times and conditions used for the SIP experiments. Table S3. Relative proportion (%) of archaeal taxa in each soil (August 2014), rock and groundwater (June, August and September 2014) sample, based on 16S rRNA gene diversi [file 2136287.f1.pdf]

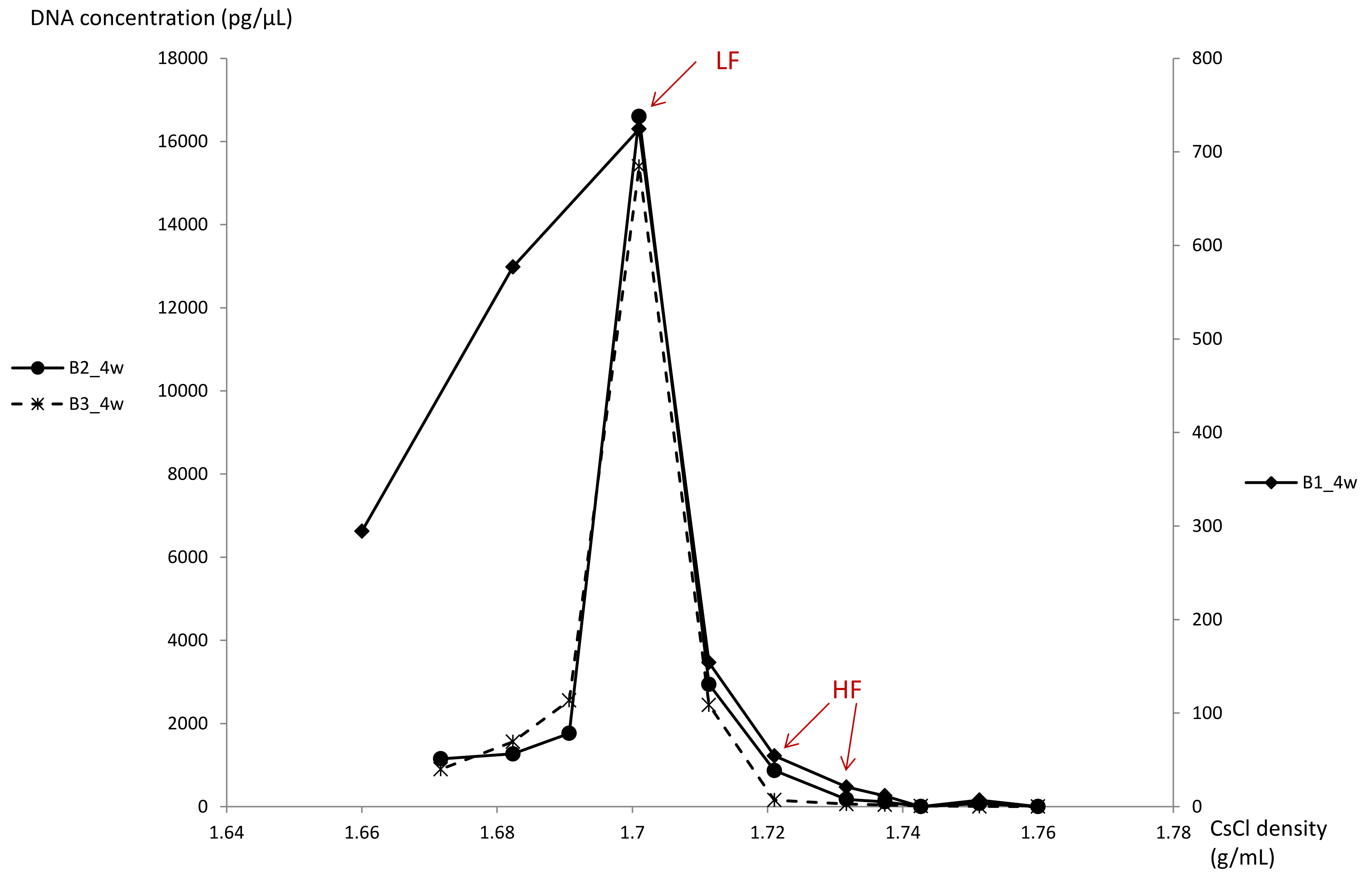
DNA concentration (pg/µL)
18000
16000
14000
12000
10000
8000
6000
4000
2000
0
800
700
600
500
400
300
200
100
0
1.64
1.66
1.68
1.7
1.72
1.74
1.76
1.78
CsCl density (g/mL)
B2_4w
B3_4w
B1_4w
LF
HF

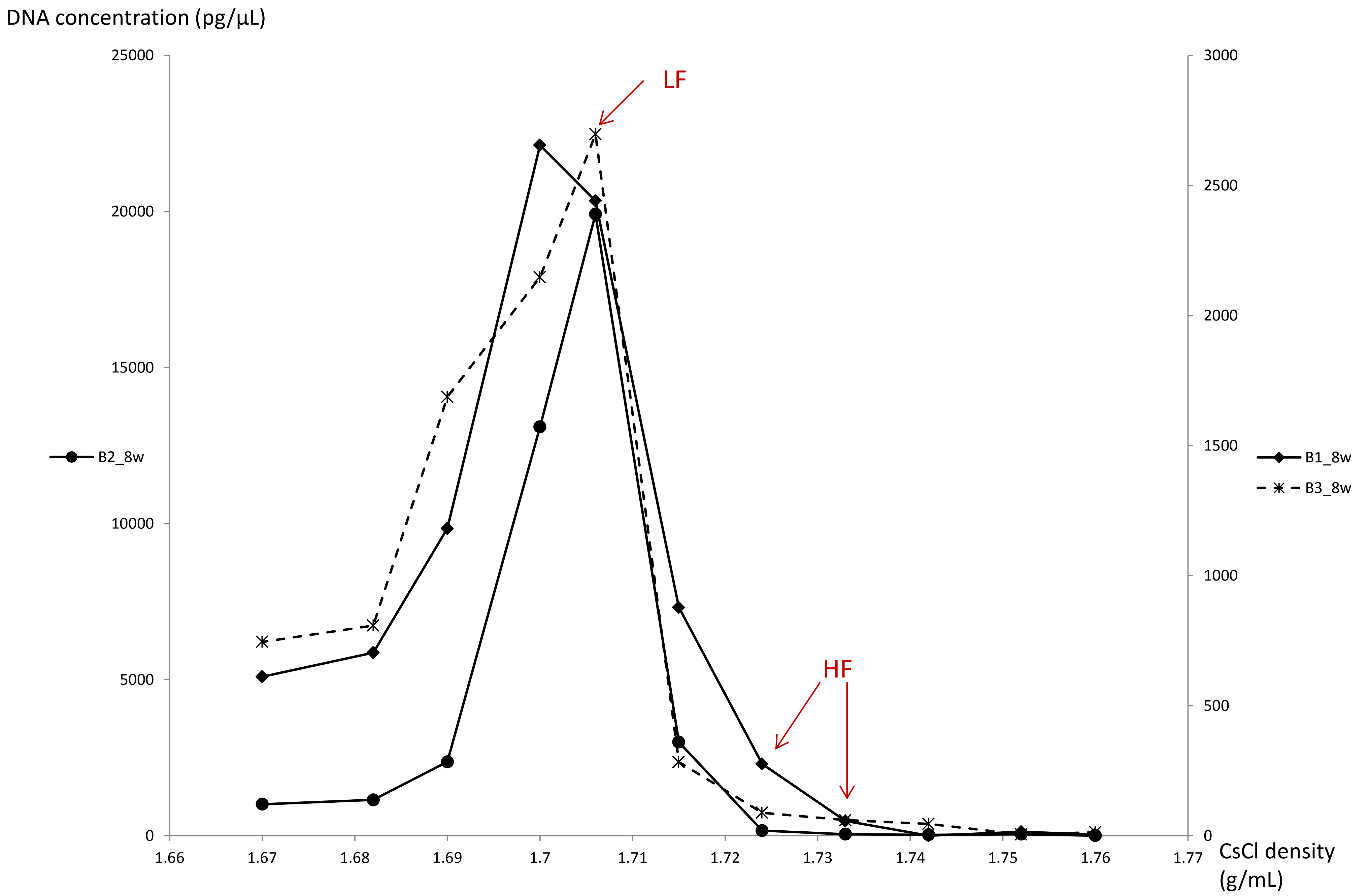
DNA concentration (pg/µL)
25000
20000
15000
10000
5000
0
3000
2500
2000
1500
1000
500
0
1.66
1.67
1.68
1.69
1.7
1.71
1.72
1.73
1.74
1.75
1.76
1.77
CsCl density (g/mL)
B2_8w
B1_8w
B3_8w
LF
HF

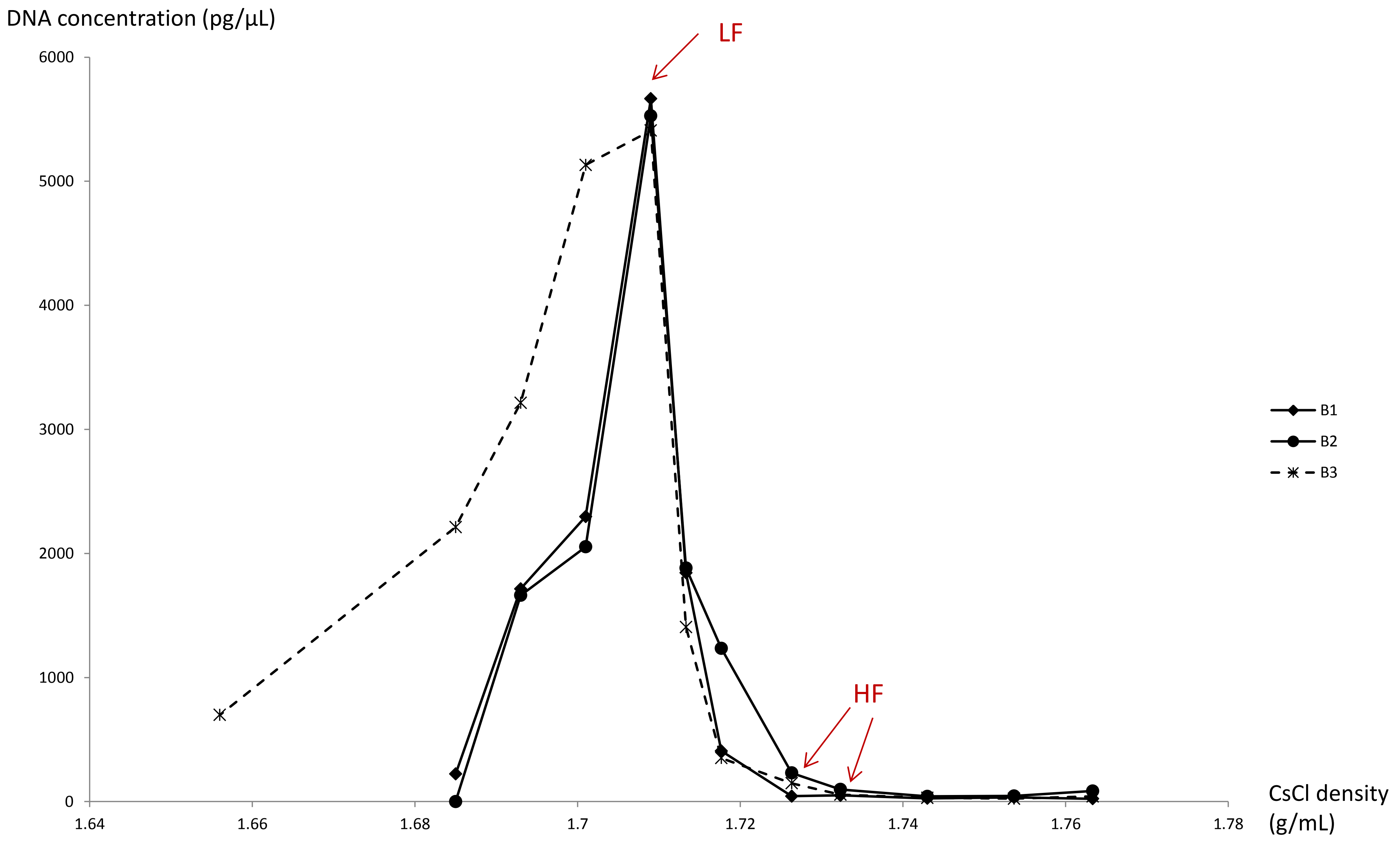
DNA concentration (pg/µL)
6000
5000
4000
3000
2000
1000
0
1.64
1.66
1.68
1.7
1.72
1.74
1.76
1.78
CsCl density (g/mL)
LF
HF
B1
B2
B3

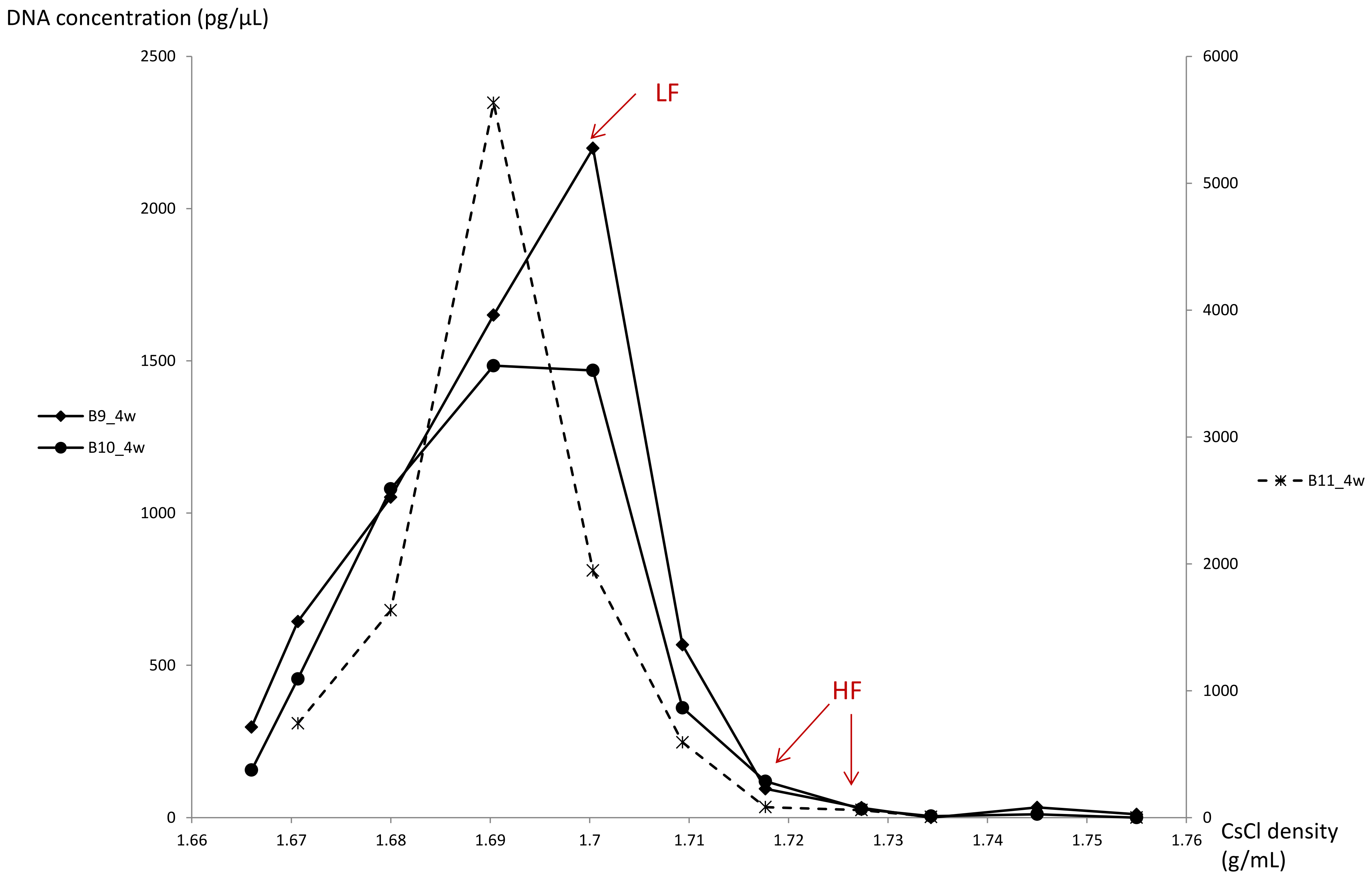
DNA concentration (pg/µL)
2500
2000
1500
1000
500
0
6000
5000
4000
3000
2000
1000
0
1.66
1.67
1.68
1.69
1.7
1.71
1.72
1.73
1.74
1.75
1.76
CsCl density (g/mL)
LF
HF
B9_4w
B10_4w
B11_4w

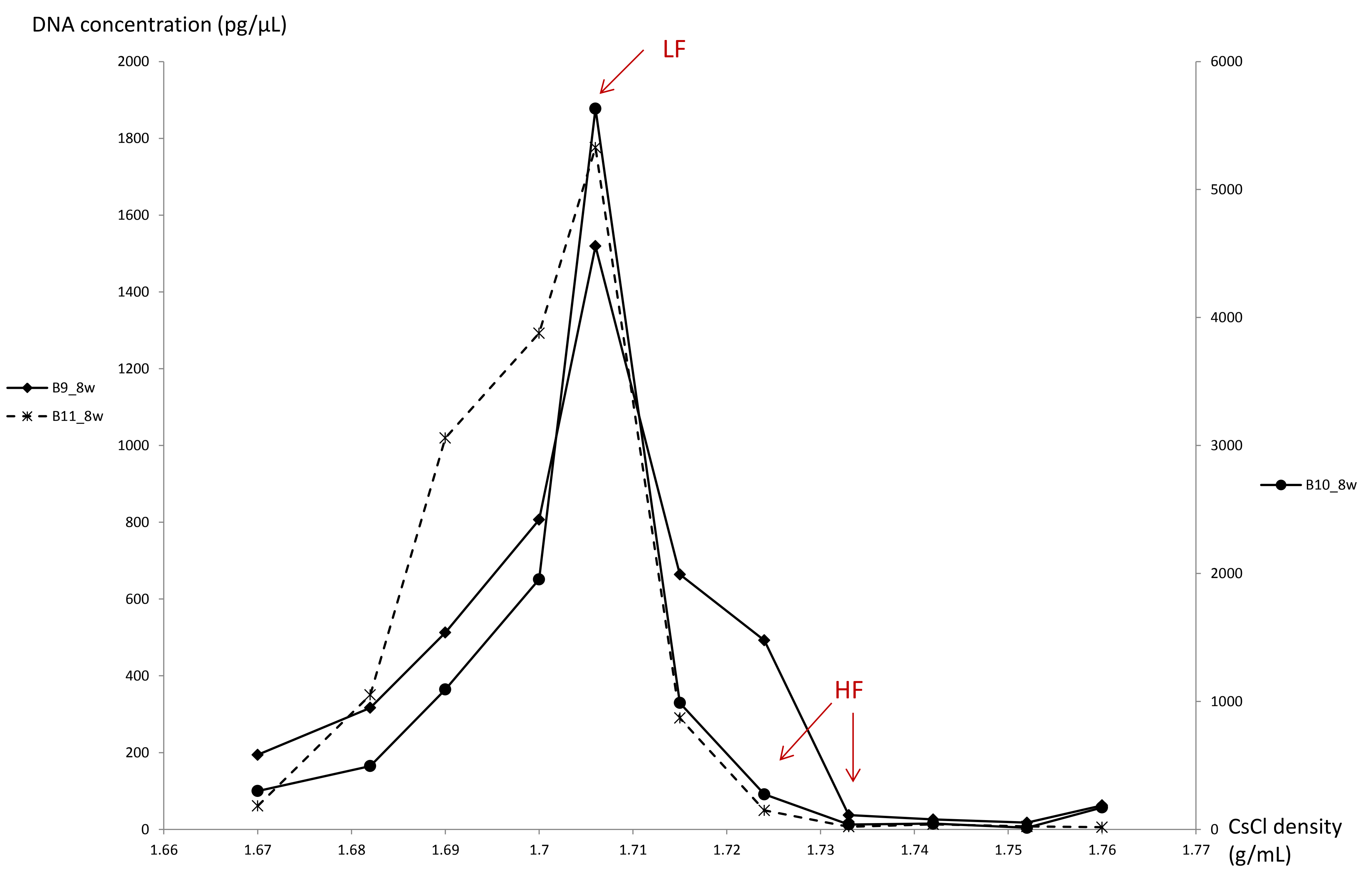
DNA concentration (pg/µL)
2000
1800
1600
1400
1200
1000
800
600
400
200
0
6000
5000
4000
3000
2000
1000
0
1.66
1.67
1.68
1.69
1.7
1.71
1.72
1.73
1.74
1.75
1.76
1.77
CsCl density (g/mL)
B9_8w
B11_8w
B10_8w
LF
HF

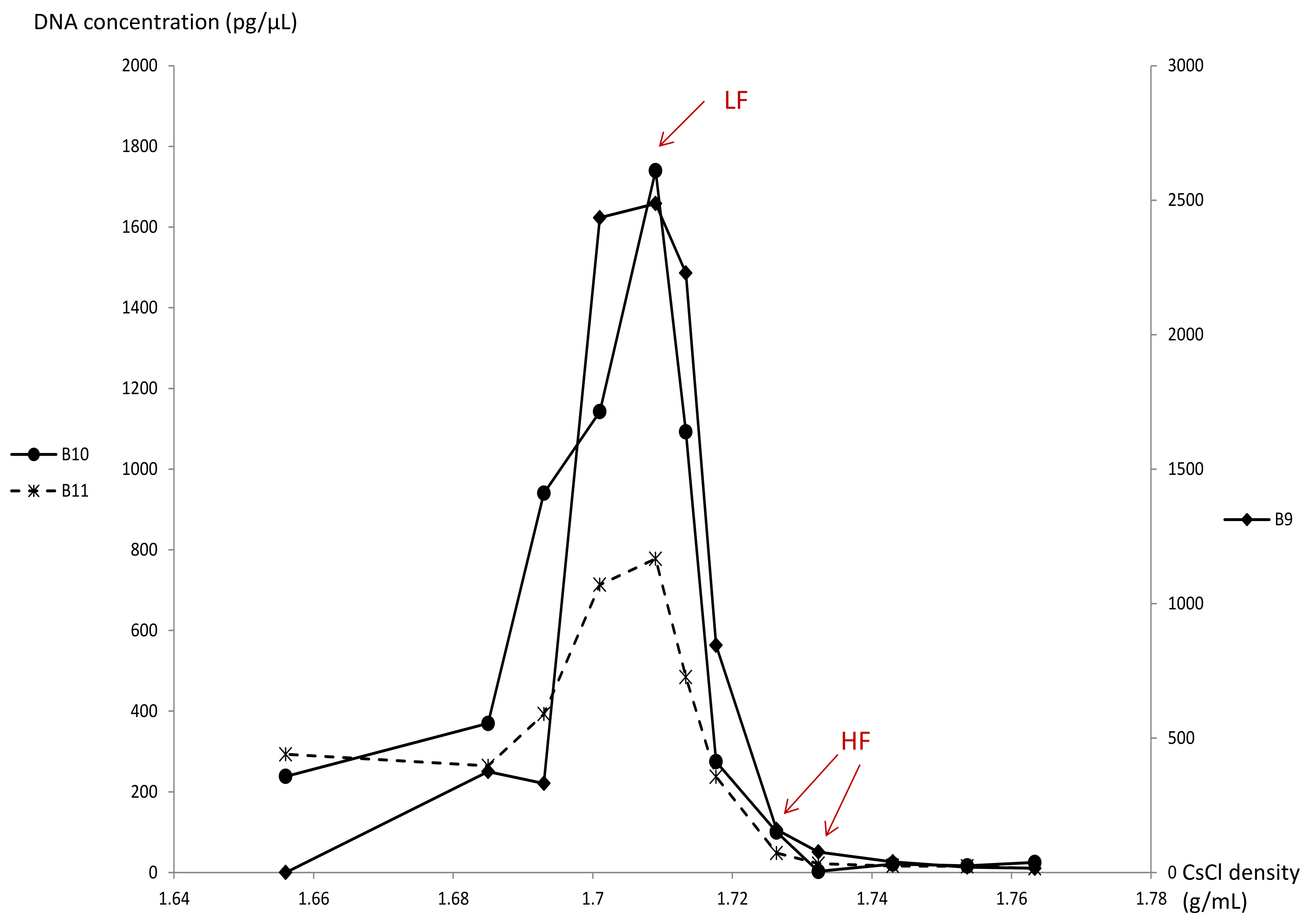

DNA concentration (pg/µL)
2000
1800
1600
1400
1200
1000
800
600
400
200
0
3000
2500
2000
1500
1000
500
0
LF
HF
B10
B11
B9
1.64
1.66
1.68
1.7
1.72
1.74
1.76
1.78
CsCl density (g/mL)

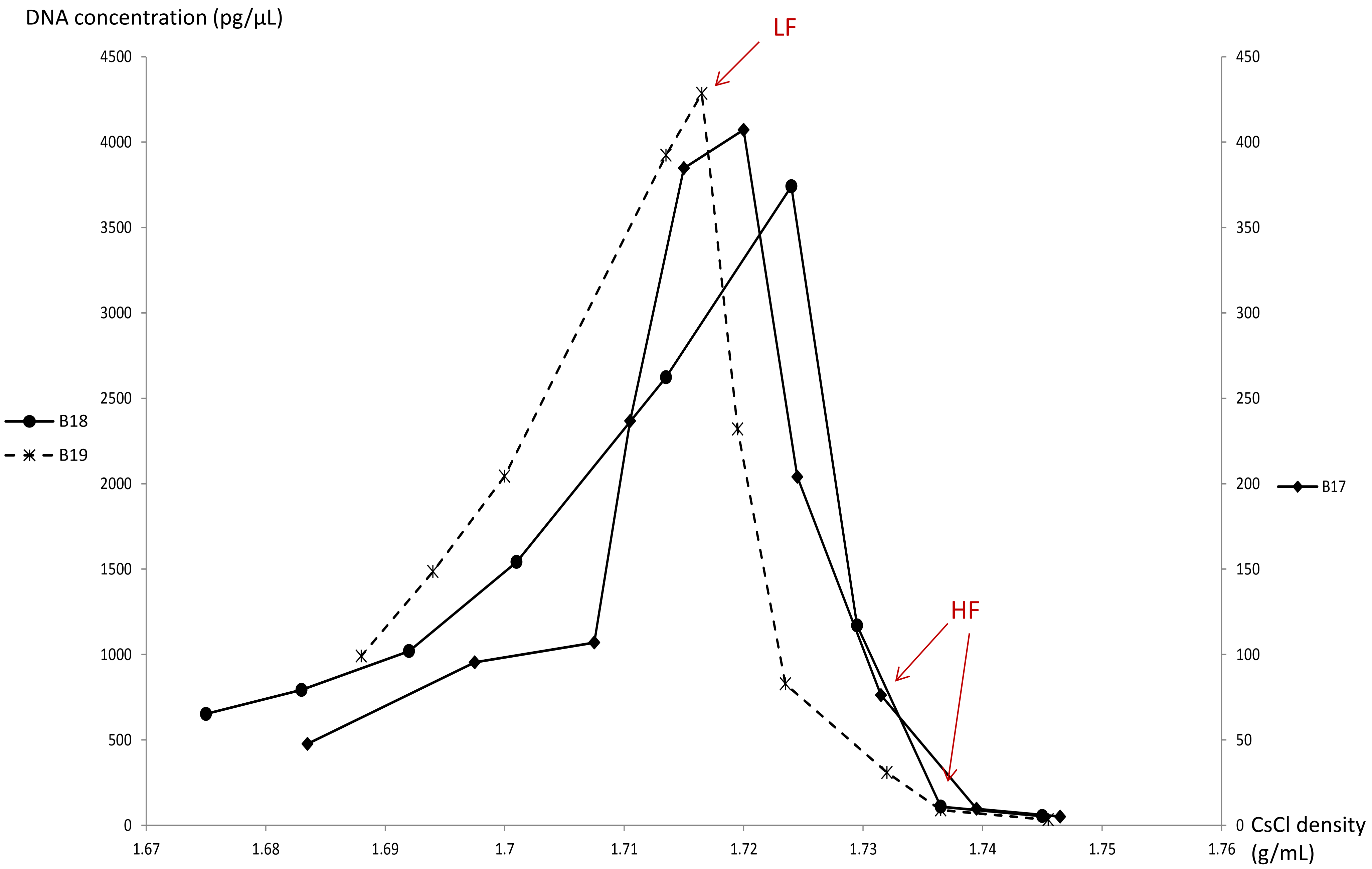

DNA concentration (pg/µL)
LF
HF
B18
B19
B17
CsCl density (g/mL)
4500
4000
3500
3000
2500
2000
1500
1000
500
0
450
400
350
300
250
200
150
100
50
0
1.67
1.68
1.69
1.7
1.71
1.72
1.73
1.74
1.75
1.76

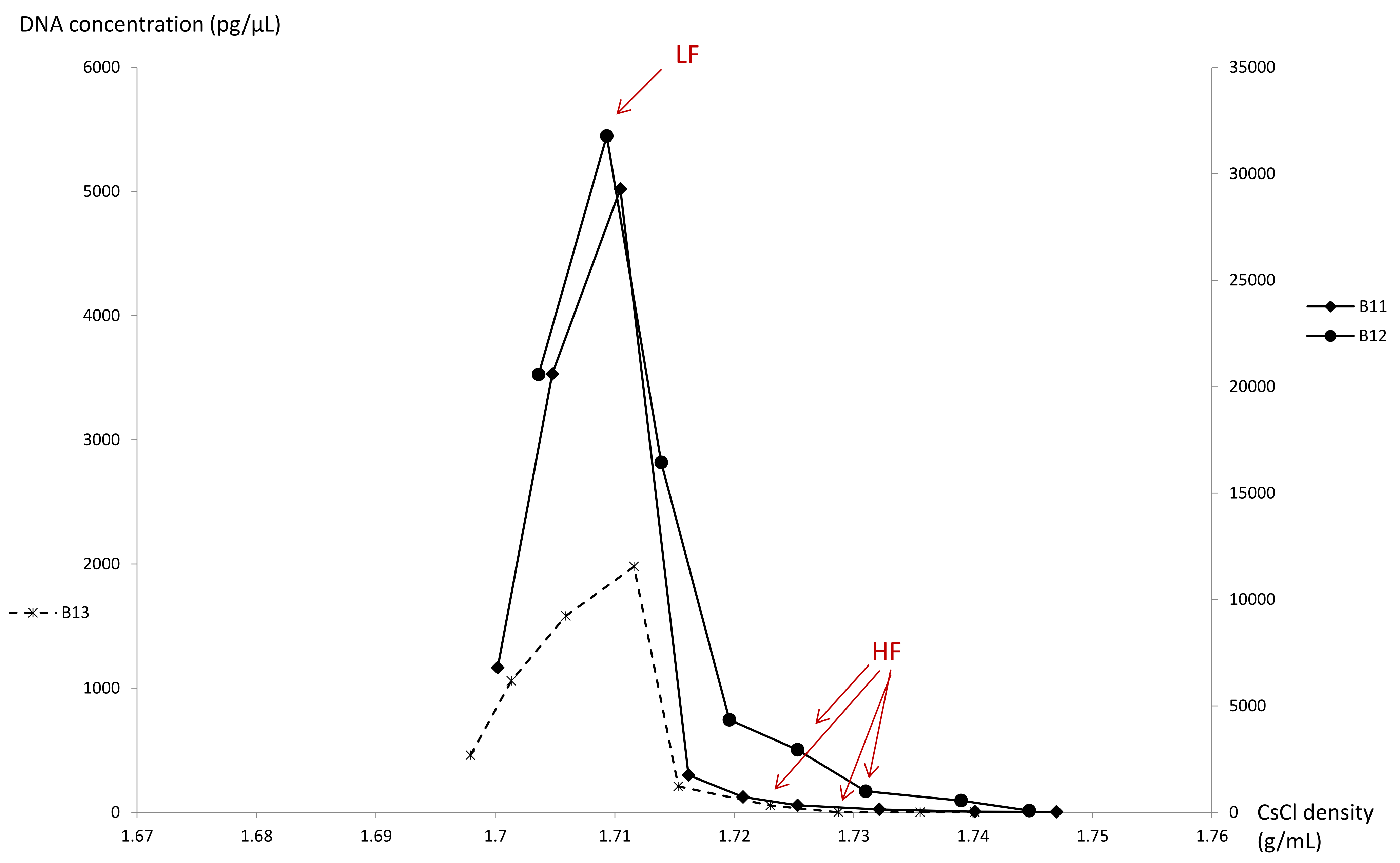
DNA concentration (pg/µL)
6000
5000
4000
3000
2000
1000
0
35000
30000
25000
20000
15000
10000
5000
0
1.67
1.68
1.69
1.7
1.71
1.72
1.73
1.74
1.75
1.76
CsCl density (g/mL)
LF
HF
B11
B12
B13

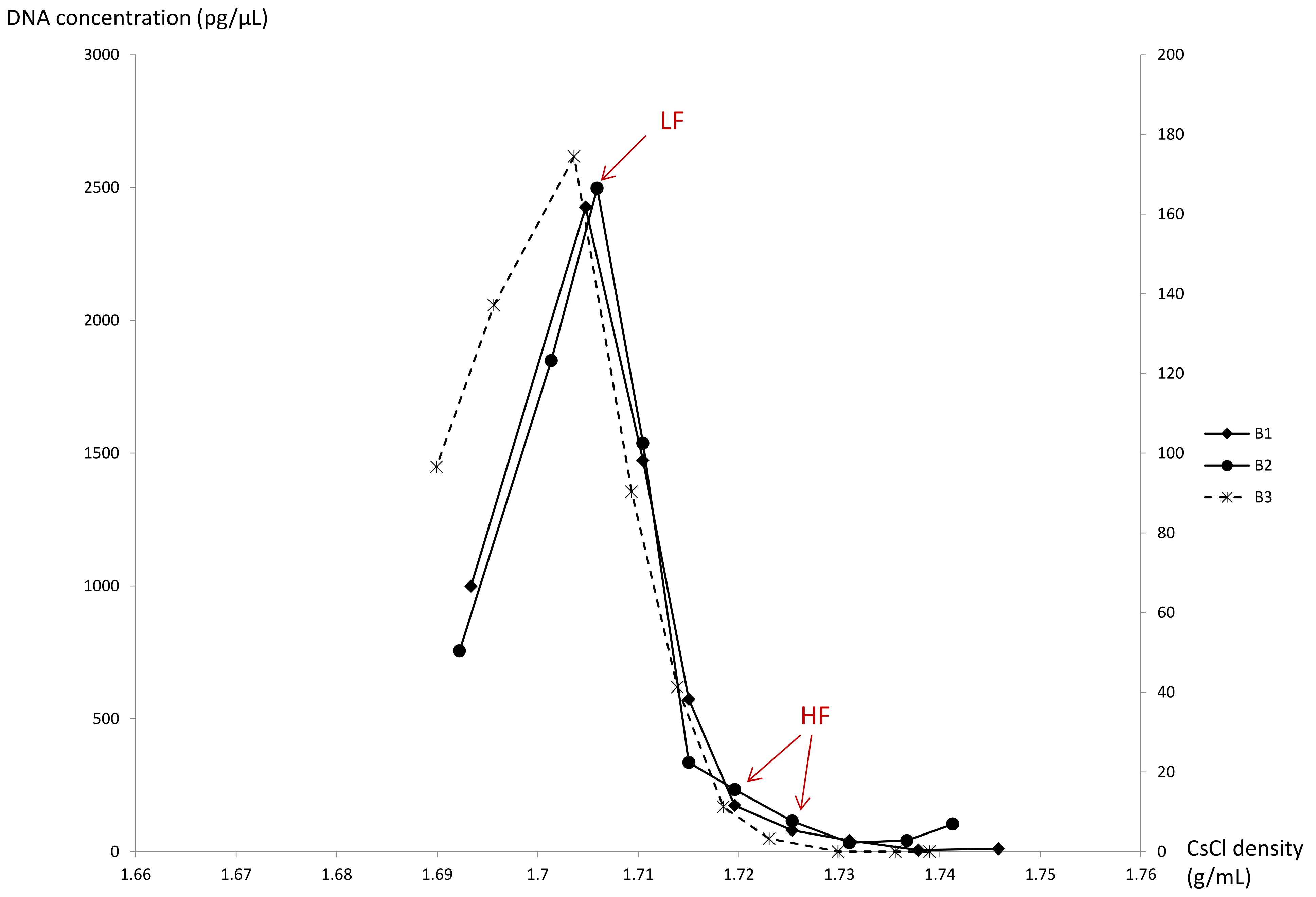

DNA concentration (pg/µL)
3000
2500
2000
1500
1000
500
0
200
180
160
140
120
100
80
60
40
20
0
1.66
1.67
1.68
1.69
1.7
1.71
1.72
1.73
1.74
1.75
1.76
CsCl density (g/mL)
LF
HF
B1
B2
B3

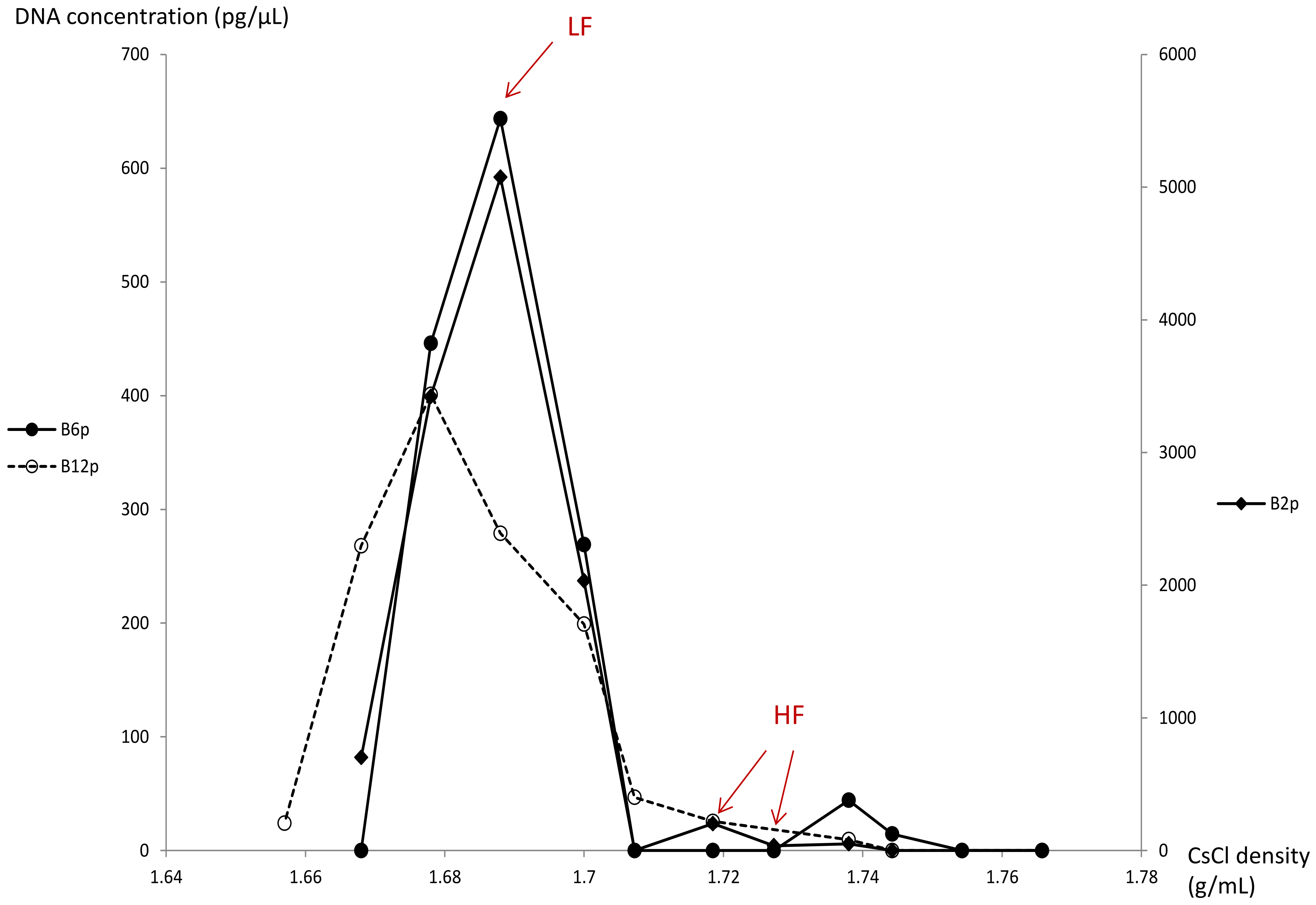

DNA concentration (pg/µL)
LF
HF
700
600
500
400
300
200
100
0
6000
5000
4000
3000
2000
1000
0
1.64
1.66
1.68
1.7
1.72
1.74
1.76
1.78
CsCl density (g/mL)
B6p
B12p
B2p

Figure S1.

(A)

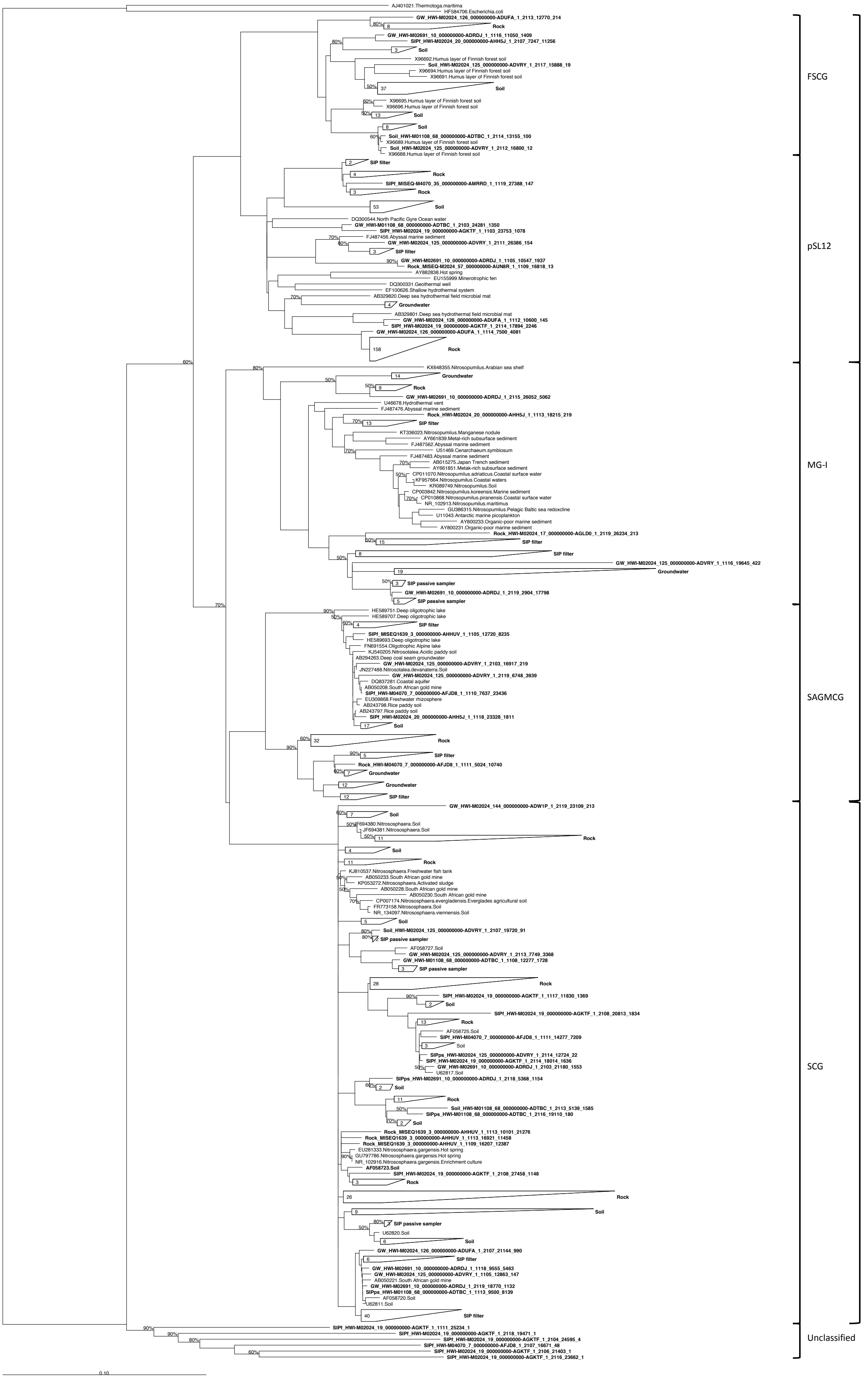

AJ401021.Thermotoga.maritima
HF584706.Escherichia.coli
FSCG
pSL12
Group 1.1c
MG-I
SAGMCG
Group 1.1a
SCG
Group 1.1b
Unclassified
0.10

(B)

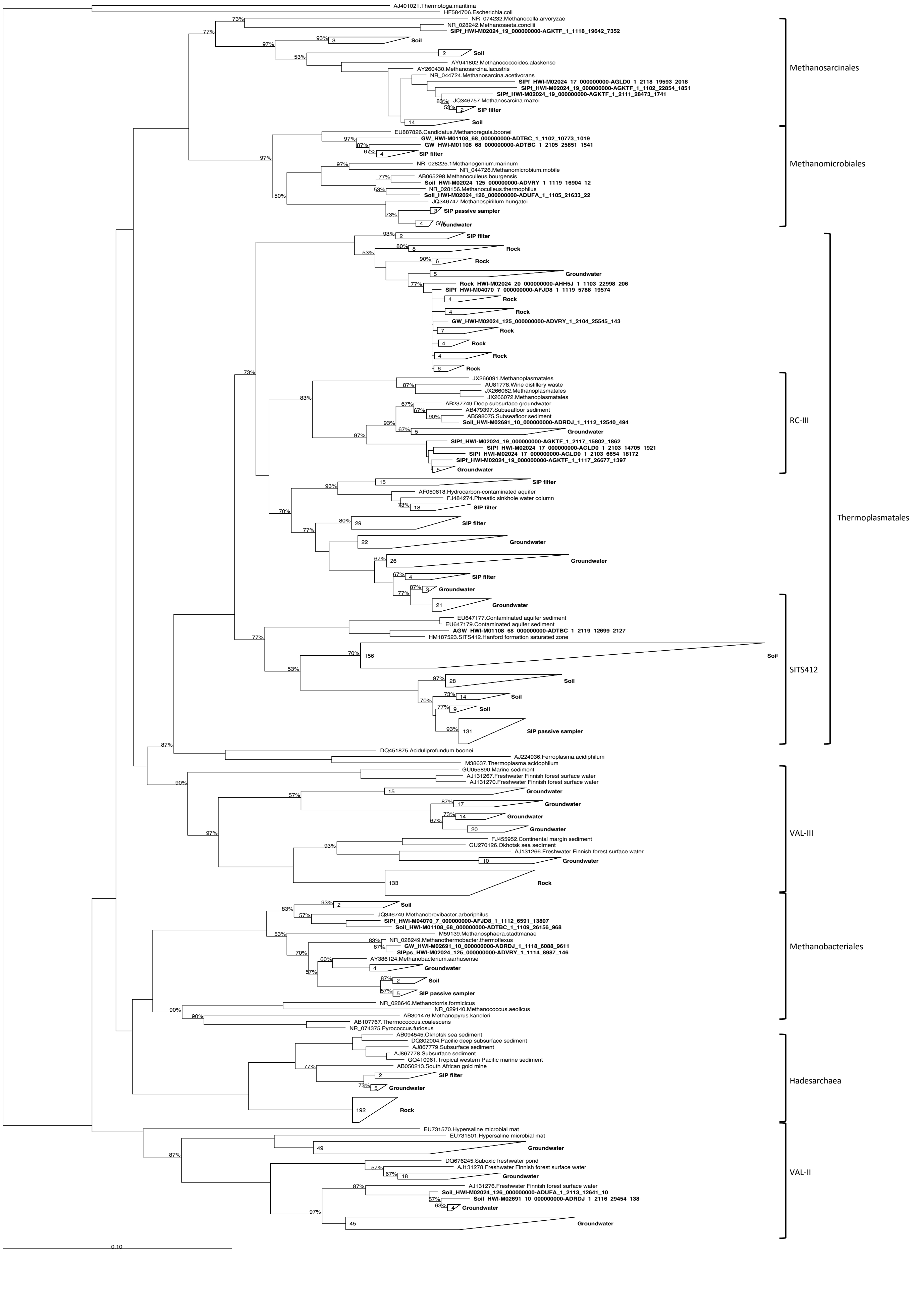
AJ401021.Thermotoga.maritima
HF584706.Escherichia.coli
NR_074232.Methanocella.arvoryzae
NR_028242.Methanosaeta.concilii
SIPf_HWI-M02024_19_000000000-AGKTF_1_1118_19642_7352
Soil
AY941802.Methanococcoides.alaskense
AY260430.Methanosarcina.lacustris
NR_044724.Methanosarcina.acetivorans
SIPf_HWI-M02024_17_000000000-AGLD0_1_2118_19593_2018
SIPf_HWI-M02024_19_000000000-AGKTF_1_1102_22854_1851
SIPf_HWI-M02024_19_000000000-AGKTF_1_2111_28473_1741
JQ346757.Methanosarcina.mazei
SIP filter
EU887826.Candidatus.Methanoregula.boonei
GW_HWI-M01108_68_000000000-ADTBC_1_1102_10773_1019
GW_HWI-M01108_68_000000000-ADTBC_1_2105_25851_1541
NR_028225.1Methanogenium.marinum
NR_044726.Methanomicrobium.mobile
AB065298.Methanoculleus.bourgensis
Soil_HWI-M02024_125_000000000-ADVRY_1_1119_16904_12
NR_028156.Methanoculleus.thermophilus
Soil_HWI-M02024_126_000000000-ADUFA_1_1105_21633_22
JQ346747.Methanospirillum.hungatei
SIP passive sampler
Groundwater
Rock
Rock_HWI-M02024_20_000000000-AHH5J_1_1103_22998_206
SIPf_HWI-M04070_7_000000000-AFJD8_1_1119_5788_19574
GW_HWI-M02024_125_000000000-ADVRY_1_2104_25545_143
JX266091.Methanoplasmatales
AU81778.Wine distillery waste
JX266062.Methanoplasmatales
JX266072.Methanoplasmatales
AB237749.Deep subsurface groundwater
AB479397.Subseafloor sediment
AB598075.Subseafloor sediment
Soil_HWI-M02691_10_000000000-ADRDJ_1_1112_12540_494
SIPf_HWI-M02024_19_000000000-AGKTF_1_2117_15802_1862
SIPf_HWI-M02024_17_000000000-AGLD0_1_2103_14705_1921
SIPf_HWI-M02024_17_000000000-AGLD0_1_2103_6654_18172
SIPf_HWI-M02024_19_000000000-AGKTF_1_1117_26677_1397
AF050618.Hydrocarbon-contaminated aquifer
FJ484274.Phreatic sinkhole water column
EU647177.Contaminated aquifer sediment
EU647179.Contaminated aquifer sediment
AGW_HWI-M01108_68_000000000-ADTBC_1_2119_12699_2127
HM187523.SITS412.Hanford formation saturated zone
DQ451875.Aciduliprofundum.boonei
AJ224936.Ferroplasma.acidiphilum
M38637.Thermoplasma.acidophilum
GU055890.Marine sediment
AJ131267.Freshwater Finnish forest surface water
AJ131270.Freshwater Finnish forest surface water
FJ455952.Continental margin sediment
GU270126.Okhotsk sea sediment
AJ131266.Freshwater Finnish forest surface water
JQ346749.Methanobrevibacter.arboriphilus
SIPf_HWI-M04070_7_000000000-AFJD8_1_1112_6591_13807
Soil_HWI-M01108_68_000000000-ADTBC_1_1109_26156_968
M59139.Methanosphaera.stadtmanae
NR_028249.Methanothermobacter.thermoflexus
GW_HWI-M02691_10_000000000-ADRDJ_1_1118_6088_9611
SIPps_HWI-M02024_125_000000000-ADVRY_1_1114_8987_146
AY386124.Methanobacterium.aarhusense
NR_028646.Methanotorris.formicicus
NR_029140.Methanococcus.aeolicus
AB301476.Methanopyrus.kandleri
AB107767.Thermococcus.coalescens
NR_074375.Pyrococcus.furiosus
AB094545.Okhotsk sea sediment
DQ302004.Pacific deep subsurface sediment
AJ867779.Subsurface sediment
AJ867778.Subsurface sediment
GQ410961.Tropical western Pacific marine sediment
AB050213.South African gold mine
EU731570.Hypersaline microbial mat
EU731501.Hypersaline microbial mat
DQ676245.Suboxic freshwater pond
AJ131278.Freshwater Finnish forest surface water
AJ131276.Freshwater Finnish forest surface water
Soil_HWI-M02024_126_000000000-ADUFA_1_2113_12641_10
Soil_HWI-M02691_10_000000000-ADRDJ_1_2116_29454_138
0.10
Methanosarcinales
Methanomicrobiales
Thermoplasmatales
RC-III
SITS412
VAL-III
Methanobacteriales
Hadesarchaea
VAL-II

(C)

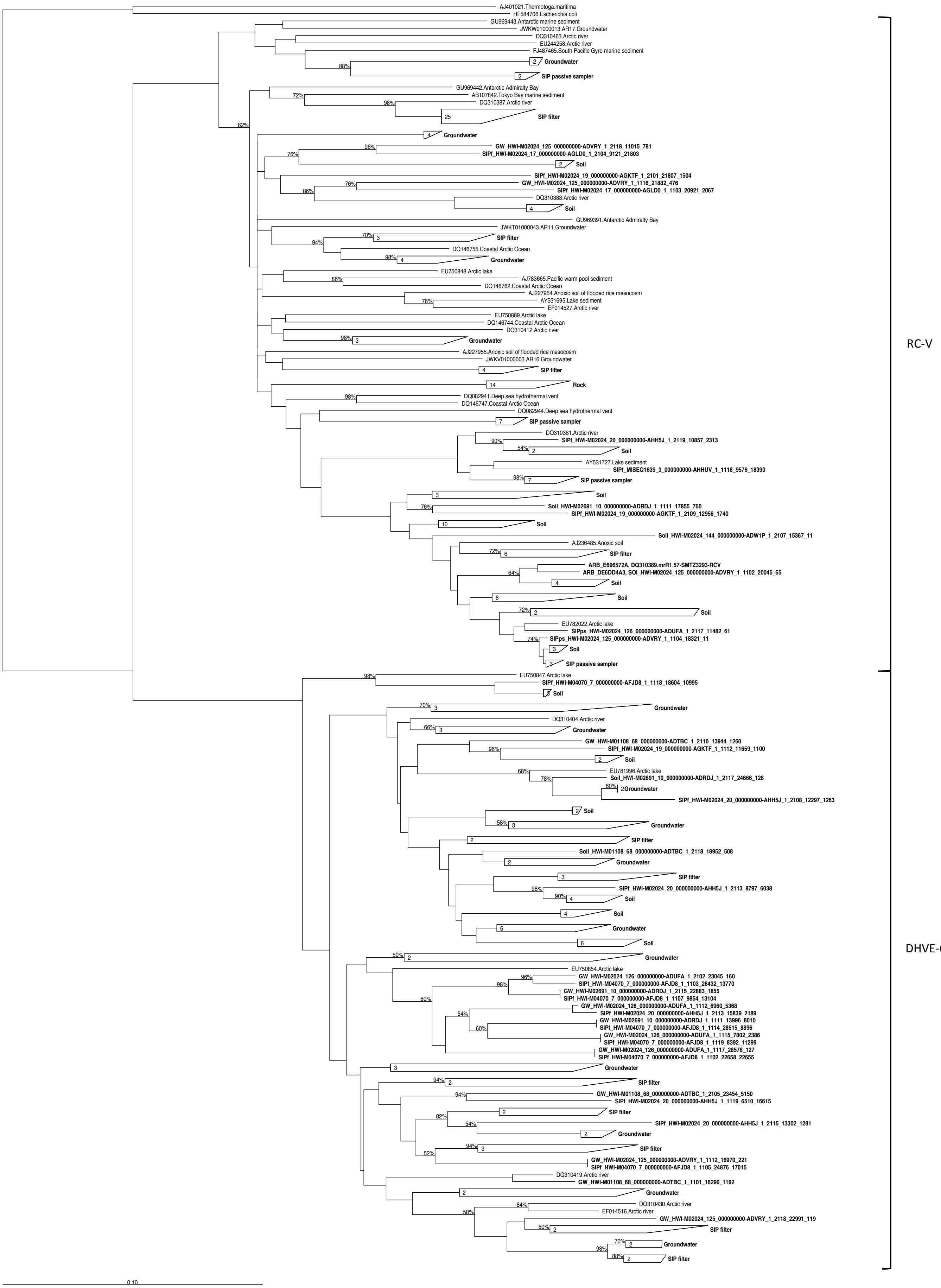

AJ401021.Thermotoga.maritima
HF584706.Escherichia.coli
RC-V
DHVE-6
Groundwater
SIP passive sampler
SIP filter
Soil
Rock
0.10

(D)

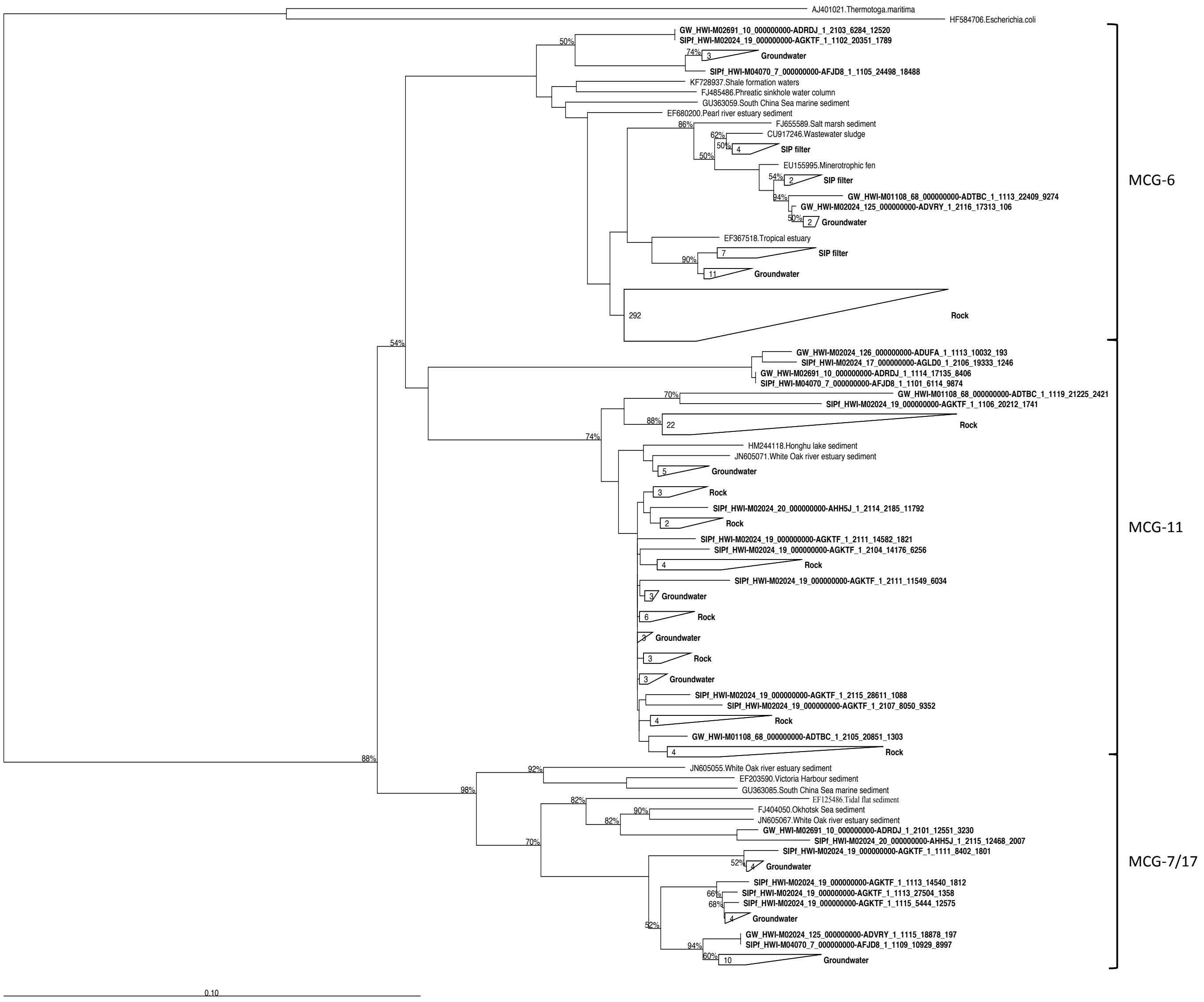

AJ401021.Thermotoga.maritima
HF584706.Escherichia.coli
GW_HWI-M02691_10_000000000-ADRDJ_1_2103_6284_12520
SIPf_HWI-M02024_19_000000000-AGKTF_1_1102_20351_1789
50%
74%
3
Groundwater
SIPf_HWI-M04070_7_000000000-AFJD8_1_1105_24498_18488
KF728937.Shale formation waters
FJ485486.Phreatic sinkhole water column
GU363059.South China Sea marine sediment
EF680200.Pearl river estuary sediment
FJ655589.Salt marsh sediment
86%
62%
CU917246.Wastewater sludge
50%
4
SIP filter
50%
EU155995.Minerotrophic fen
54%
2
SIP filter
94%
GW_HWI-M01108_68_000000000-ADTBC_1_1113_22409_9274
GW_HWI-M02024_125_000000000-ADVRY_1_2116_17313_106
50%
2
Groundwater
EF367518.Tropical estuary
90%
7
SIP filter
11
Groundwater
292
Rock
54%
GW_HWI-M02024_126_000000000-ADUFA_1_1113_10032_193
SIPf_HWI-M02024_17_000000000-AGLD0_1_2106_19333_1246
GW_HWI-M02691_10_000000000-ADRDJ_1_1114_17135_8406
SIPf_HWI-M04070_7_000000000-AFJD8_1_1101_6114_9874
70%
GW_HWI-M01108_68_000000000-ADTBC_1_1119_21225_2421
SIPf_HWI-M02024_19_000000000-AGKTF_1_1106_20212_1741
88%
22
Rock
74%
HM244118.Honghu lake sediment
JN605071.White Oak river estuary sediment
5
Groundwater
3
Rock
SIPf_HWI-M02024_20_000000000-AHH5J_1_2114_2185_11792
2
Rock
SIPf_HWI-M02024_19_000000000-AGKTF_1_2111_14582_1821
SIPf_HWI-M02024_19_000000000-AGKTF_1_2104_14176_6256
4
Rock
SIPf_HWI-M02024_19_000000000-AGKTF_1_2111_11549_6034
3
Groundwater
6
Rock
3
Groundwater
3
Rock
3
Groundwater
SIPf_HWI-M02024_19_000000000-AGKTF_1_2115_28611_1088
SIPf_HWI-M02024_19_000000000-AGKTF_1_2107_8050_9352
4
Rock
GW_HWI-M01108_68_000000000-ADTBC_1_2105_20851_1303
4
Rock
88%
92%
JN605055.White Oak river estuary sediment
98%
EF203590.Victoria Harbour sediment
GU363085.South China Sea marine sediment
82%
EF125486.Tidal flat sediment
90%
FJ404050.Okhotsk Sea sediment
82%
JN605067.White Oak river estuary sediment
GW_HWI-M02691_10_000000000-ADRDJ_1_2101_12551_3230
70%
SIPf_HWI-M02024_20_000000000-AHH5J_1_2115_12468_2007
SIPf_HWI-M02024_19_000000000-AGKTF_1_1111_8402_1801
52%
4
Groundwater
SIPf_HWI-M02024_19_000000000-AGKTF_1_1113_14540_1812
66%
SIPf_HWI-M02024_19_000000000-AGKTF_1_1113_27504_1358
68%
SIPf_HWI-M02024_19_000000000-AGKTF_1_1115_5444_12575
4
Groundwater
52%
GW_HWI-M02024_125_000000000-ADVRY_1_1115_18878_197
94%
SIPf_HWI-M04070_7_000000000-AFJD8_1_1109_10929_8997
60%
10
Groundwater
0.10
MCG-6
MCG-11
MCG-7/17

Figure S2.

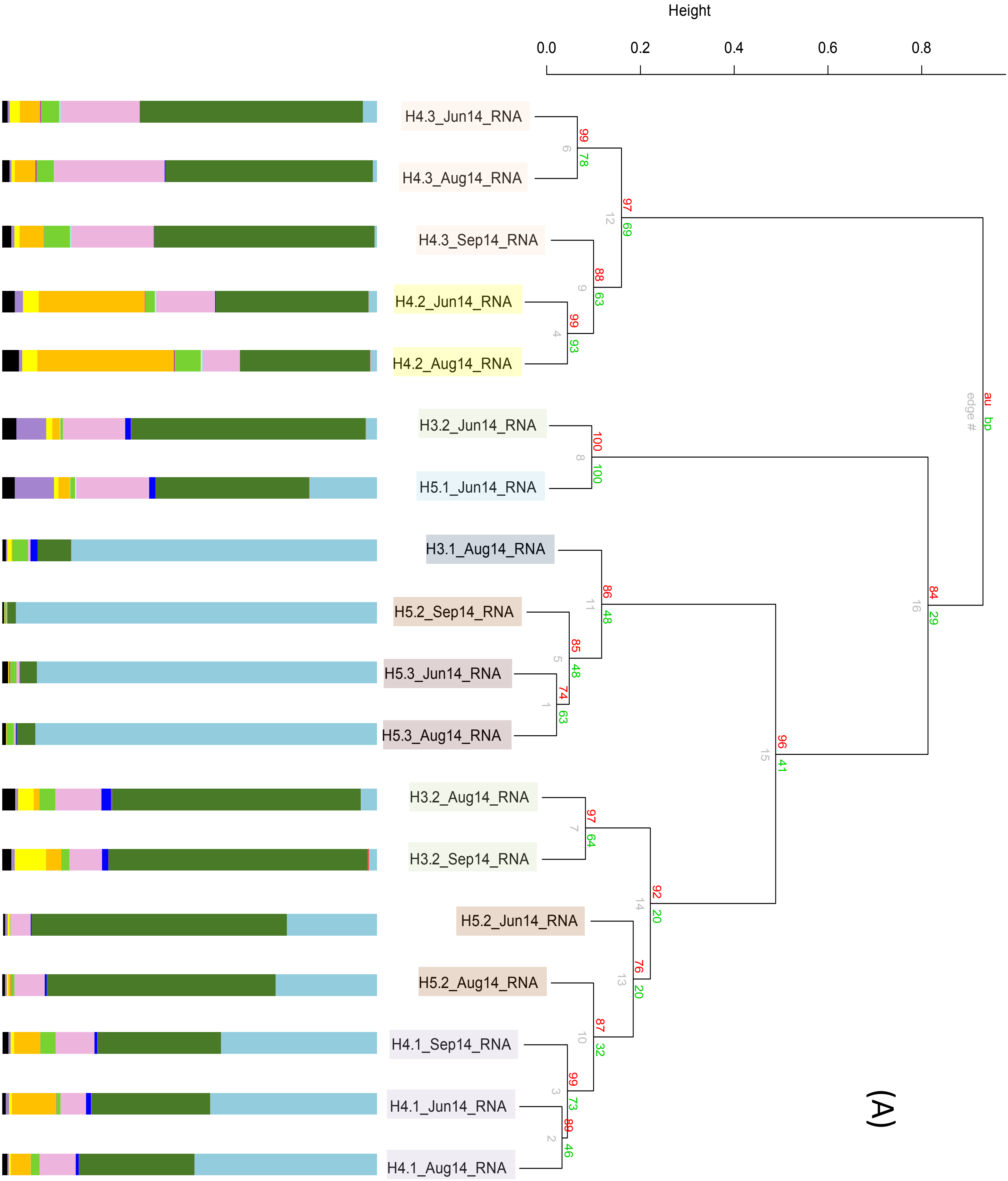
Height
0.0
0.2
0.4
0.6
0.8
H4.3_Jun14_RNA
H4.3_Aug14_RNA
H4.3_Sep14_RNA
H4.2_Jun14_RNA
H4.2_Aug14_RNA
H3.2_Jun14_RNA
H5.1_Jun14_RNA
H3.1_Aug14_RNA
H5.2_Sep14_RNA
H5.3_Jun14_RNA
H5.3_Aug14_RNA
H3.2_Aug14_RNA
H3.2_Sep14_RNA
H5.2_Jun14_RNA
H5.2_Aug14_RNA
H4.1_Sep14_RNA
H4.1_Jun14_RNA
H4.1_Aug14_RNA
au
bp
edge #
(A)

Figure S3.

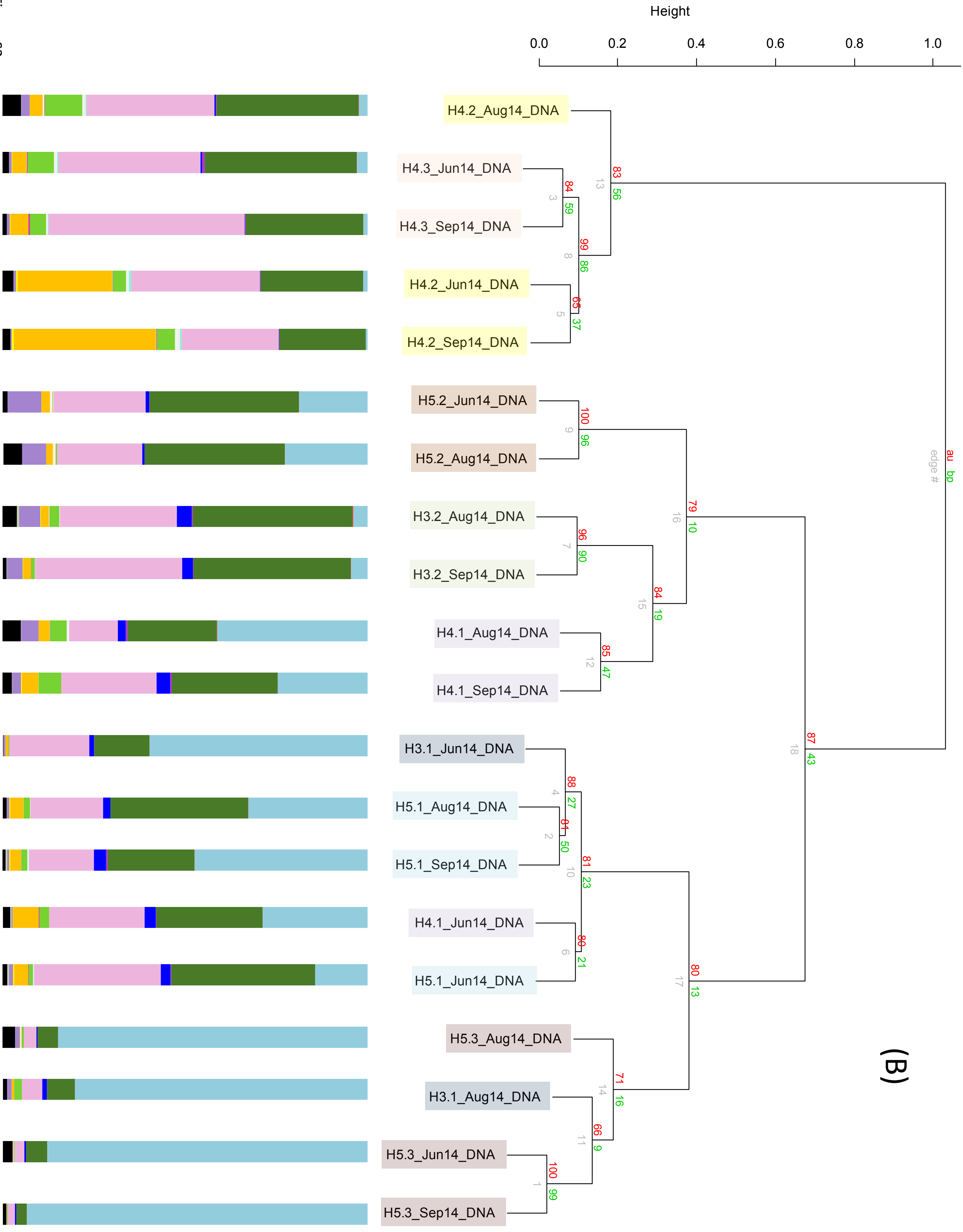

Height
0.0
0.2
0.4
0.6
0.8
1.0
H4.2_Aug14_DNA
H4.3_Jun14_DNA
H4.3_Sep14_DNA
H4.2_Jun14_DNA
H4.2_Sep14_DNA
H5.2_Jun14_DNA
H5.2_Aug14_DNA
H3.2_Aug14_DNA
H3.2_Sep14_DNA
H4.1_Aug14_DNA
H4.1_Sep14_DNA
H3.1_Jun14_DNA
H5.1_Aug14_DNA
H5.1_Sep14_DNA
H4.1_Jun14_DNA
H5.1_Jun14_DNA
H5.3_Aug14_DNA
H3.1_Aug14_DNA
H5.3_Jun14_DNA
H5.3_Sep14_DNA
au
bp
edge #
(B)

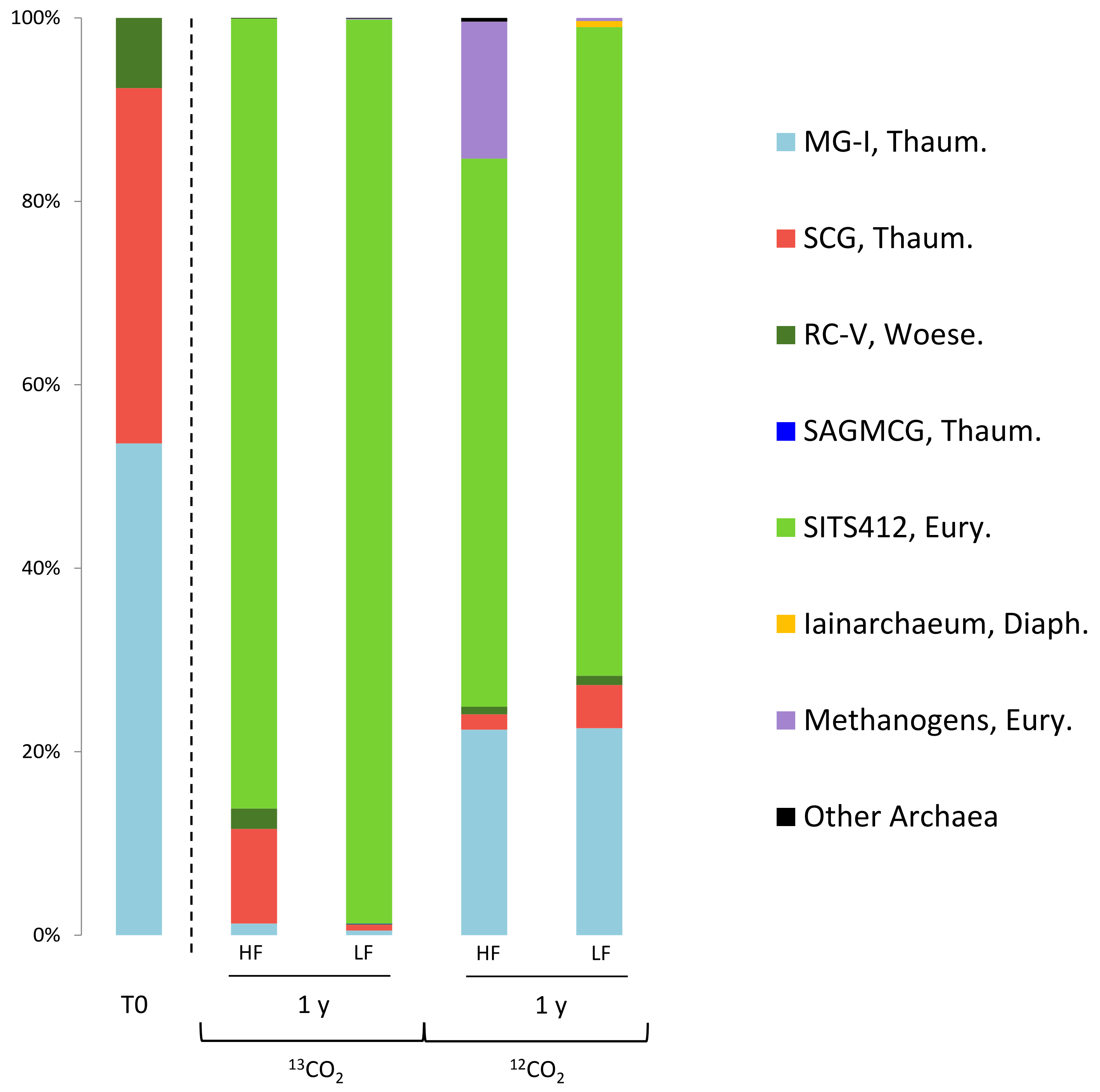
100%
80%
60%
40%
20%
0%
HF
LF
HF
LF
T0
1 y
1 y
$^{13}CO_2$
$^{12}CO_2$
MG-I, Thaum.
SCG, Thaum.
RC-V, Woese.
SAGMCG, Thaum.
SITS412, Eury.
Iainarchaeum, Diaph.
Methanogens, Eury.
Other Archaea

Figure S4.

**Rock core sample ID**	**Site**	**Well**	**Depth (drilling meter below surface)**	**Lithostratigraphic unit**	**Rock type (limestone type)**	**Aquifer type**
**KS-20**	H3	H3-1	1.70 - 1.80	Quarternary	Loess	Aquitard
**KS-38**	H3	H3-2	12.5 – 12.72	Upper Muschelkalk, Meissner formation	Limestone (grainstone)	Joint aquifer
**KS-41**	H5	H5-2/3	5.40 – 5.50	Lower Keuper, Erfurt formation	Siltstone	Aquitard
**KS-3**	H5	H5-1	7.24 – 7.35	Lower Keuper, Erfurt formation	Limestone-marlstone (mudstone)	Aquitard
**KS-8**	H5	H5-1	21.30 – 21.60	Lower Keuper, Erfurt formation	Silty sandstone, dolomitic	Porous aquifer
**KS-51**	H5	H5-2/3	50.40 – 50.53	Upper Muschelkalk, Meissner formation	Limestone (wackestone)	Joint aquifer

Table S1.

Well sampled	Material incubated	Substrate used for incubation	Time of incubation	Incubation conditions	Sample ID (bottle #)	# fractions obtained
H4-3	Filter	$^{13}C-CO_2$	1 month	Anoxic	B1_4w (Duplicate 1)	11
H4-3	Filter	$^{13}C-CO_2$	1 month	Anoxic	B2_4w (Duplicate 2)	11
H4-3	Filter	$^{12}C-CO_2$	1 month	Anoxic	B3_4w (Control)	11
H4-3	Filter	$^{13}C-CO_2$	2 months	Anoxic	B1_8w (Duplicate 1)	11
H4-3	Filter	$^{13}C-CO_2$	2 months	Anoxic	B2_8w (Duplicate 2)	11
H4-3	Filter	$^{12}C-CO_2$	2 months	Anoxic	B3_8w (Control)	11
H4-3	Filter	$^{13}C-CO_2$	3 months	Anoxic	B1 (Duplicate 1)	12
H4-3	Filter	$^{13}C-CO_2$	3 months	Anoxic	B2 (Duplicate 2)	12
H4-3	Filter	$^{12}C-CO_2$	3 months	Anoxic	B3 (Control)	12
H4-1	Filter	$^{13}C-CO_2$	1 month	Oxic	B9_4w (Duplicate 1)	11
H4-1	Filter	$^{13}C-CO_2$	1 month	Oxic	B10_4w (Duplicate 2)	11
H4-1	Filter	$^{12}C-CO_2$	1 month	Oxic	B11_4w (Control)	11
H4-1	Filter	$^{13}C-CO_2$	2 months	Oxic	B9_8w (Duplicate 1)	11
H4-1	Filter	$^{13}C-CO_2$	2 months	Oxic	B10_8w (Duplicate 2)	11
H4-1	Filter	$^{12}C-CO_2$	2 months	Oxic	B11_8w (Control)	11
H4-1	Filter	$^{13}C-CO_2$	3 months	Oxic	B9 (Duplicate 1)	12
H4-1	Filter	$^{13}C-CO_2$	3 months	Oxic	B10 (Duplicate 2)	12
H4-1	Filter	$^{12}C-CO_2$	3 months	Oxic	B11 (Control)	12
H4-1	Filter	^{13}C-Veratric acid	3 months	Oxic	B17 (Duplicate 1)	11
H4-1	Filter	^{13}C-Veratric acid	3 months	Oxic	B18 (Duplicate 2)	11
H4-1	Filter	^{12}C-Veratric acid	3 months	Oxic	B19 (Control)	11
H4-1	Passive sampler	$^{13}C-CO_2$	1 year	Oxic	B2p (Duplicate 1)	11
H4-1	Passive sampler	$^{13}C-CO_2$	1 year	Oxic	B6p (Duplicate 2)	11
H4-1	Passive sampler	$^{12}C-CO_2$	1 year	Oxic	B12p (Control)	11
H5-2	Filter	$^{13}C-CO_2$	5 weeks	Anoxic	B11_H5 (Duplicate 1)	12
H5-2	Filter	$^{13}C-CO_2$	5 weeks	Anoxic	B12_H5 (Duplicate 2)	12
H5-2	Filter	$^{12}C-CO_2$	5 weeks	Anoxic	B13_H5 (Control)	12
H5-1	Filter	$^{13}C-CO_2$	5 weeks	Oxic	B1_H5 (Duplicate 1)	12
H5-1	Filter	$^{13}C-CO_2$	5 weeks	Oxic	B2_H5 (Duplicate 2)	12
H5-1	Filter	$^{12}C-CO_2$	5 weeks	Oxic	B3_H5 (Control)	12

Table S2.

	Top soil samples							
	H1		H2a		H2b		H3	
	**RNA** (%)	**DNA** (%)	**RNA** (%)	**DNA** (%)	**RNA** (%)	**DNA** (%)	**RNA** (%)	**DNA** (%)
**FSCG**	n.a.	96.83	28.33	79.6	0	0.04	0	0.01
**SCG**	n.a.	0.45	59.38	3.65	99.87	97.72	99.94	99.14
**MG-I**	n.a.	0	0	0.04	0.01	0	0	0
**RC-V**	n.a.	1.59	3.91	4.07	0.05	0.84	0.02	0.12
**DHVE-6**	n.a.	0	0	0	0.03	0.42	0	0.03
**Cand. Iainarchaeum**	n.a.	0.23	0	0	0	0	0	0
**SITS412**	n.a.	0.11	7.74	12.35	0.05	0.52	0.04	0.12
**Hadesarchaea**	n.a.	0.45	0	0.19	0	0.39	0	0
**pSL12**	n.a.	0.06	0	0	0	0	0	0
**VAL-III**	n.a.	0	0	0	0.01	0	0	0
**Methanogens**	n.a.	0.17	0.4	0.08	0	0.06	0	0.57
**VAL-II**	n.a.	0.11	0.24	0.04	0	0.01	0	0
**Total # sequences**	258	1766	1257	2635	19982	14223	35985	23694

	Rock core samples					
	**H3_1.7-1.8 m Aquitard DNA** (%)	**H3_12.5-12.72 m Joint aquifer DNA** (%)	**H5_3-5.4-5.5 m Aquitard DNA** (%)	**H5_7.24-7.35 m Aquitard DNA** (%)	**H5_21.16-21.3 m Porous aquifer DNA** (%)	**H5_50.4-50.53 m Joint aquifer DNA** (%)
**FSCG**	0.66	0	0	0	0	0
**SCG**	91.21	0	0.87	0.58	0.19	77.9
**MG-I**	1.33	0.09	0.01	0	0	0
**RC-V**	0	0	0	0.04	0	0
**SITS412**	0.09	0	2.86	0	0	0
**Hadesarchaea**	0	0	0.27	0	0	21.54
**MBG-A**	0.08	0	0.24	56.43	25.07	0
**pSL12**	0.28	0	34.06	2.65	1.96	0
**SAGMCG**	5.13	99.91	0.02	0	0	0
**MCG-6**	0	0	0	39.66	0	0
**MCG-11**	0	0	0	0	72.03	0
**VAL-III**	0	0	12.32	0	0	0.01
**Methanogens**	0	0	0.34	0	0	0.17
**DSEG**	0	0	39.49	0	0	0
**MBG-E**	0	0	8.57	0	0	0
**Group_1.1c**	0	0	0.2	0.22	0.59	0
**Unclassified**	1.22	0	0.75	0.42	0.16	0.38
**Total # sequences**	11802	77103	50566	12551	61237	86725

	Groundwater samples – August 2014							
	H3-1		H4-1		H5-1		H3-2	
	**RNA** (%)	**DNA** (%)	**RNA** (%)	**DNA** (%)	**RNA** (%)	**DNA** (%)	**RNA** (%)	**DNA** (%)
**FSCG**	0	0.02	0	0.1	n.a.	0.04	0.14	0.61
**SCG**	0.09	0.06	0	0.13	n.a.	0.04	0.02	0.21
**MG-I**	81.71	80.29	48.85	41.36	n.a.	32.94	4.94	4.33
**RC-V**	8.87	7.6	30.74	24.33	n.a.	37.6	65.81	43.56
**DHVE-6**	0.68	5.57	9.58	13.27	n.a.	19.82	12.11	31.92
**Cand. Iainarchaeum**	0.21	0.67	5.26	2.99	n.a.	3.6	1.58	2.16
**SITS412**	4.17	2.06	2.35	4.54	n.a.	1.76	4.2	2.67
**Hadesarchaea**	0	0.02	0.03	0.07	n.a.	0.04	0	0.16
**MBG-A**	0	0	0	0.4	n.a.	0	0.04	0
**pSL12**	0	0.04	0.05	0.13	n.a.	0	0.27	0.33
**SAGMCG**	1.87	1.26	0.81	2.12	n.a.	2.04	2.55	4.02
**MCG-6**	0	0	0	0	n.a.	0.04	0.05	0
**MCG-11**	0	0	0	0.67	n.a.	0.08	0	0.14
**VAL-III**	1.1	0.02	0.52	0	n.a.	0.25	4.09	0.16
**Methanogens**	0.34	1.21	0.39	4.74	n.a.	0.69	0.59	5.79
**DSEG**	0	0.04	0.03	0.07	n.a.	0	0.02	0.03
**MBG-E**	0	0	0.47	0.87	n.a.	0	0.26	0.21
**MG-IV**	0	0.02	0	0.34	n.a.	0	0.04	0.34
**VAL-II**	0.71	0.67	0.65	0.74	n.a.	0.37	2.93	0.89
**RC-III**	0.21	0	0.21	0.64	n.a.	0	0	0
**Group_1.1c**	0	0.17	0	0.44	n.a.	0.16	0.02	0.95
**Unclassified**	0.04	0.28	0.06	2.05	0	0.53	0.34	1.52
**Total # sequences**	3258	4617	3822	2976	182	2447	9350	7046

	Groundwater samples – August 2014							
	**H4-2**		**H4-3**		**H5-2**		**H5-3**	
	**RNA** (%)	**DNA** (%)	**RNA** (%)	**DNA** (%)	**RNA** (%)	**DNA** (%)	**RNA** (%)	**DNA** (%)
**FSCG**	0	0.07	0	n.a.	0	0	0	0
**SCG**	0.12	0.11	0	n.a.	0.01	0.04	0	0
**MG-I**	1.98	2.35	1.56	n.a.	27.25	23.07	91.11	84.78
**RC-V**	34.68	38.74	55.01	n.a.	60.92	38.32	4.96	5.61
**DHVE-6**	9.98	34.89	29.31	n.a.	8.08	23.32	0.45	3.44
**Cand. Iainarchaeum**	36.37	3.46	5.44	n.a.	0.64	1.84	0.14	0.13
**SITS412**	6.95	10.2	4.6	n.a.	0.92	0.35	1.9	0.59
**Hadesarchaea**	0	0.46	0	n.a.	0	0.74	0	0.39
**MBG-A**	0	0.14	0.02	n.a.	0	0	0	0
**SAGMCG**	0	0.39	0.23	n.a.	0.52	0.64	0.23	0.31
**MCG-6**	0.29	0.25	0	n.a.	0	0	0	0
**MCG-11**	0.18	0.89	0.05	n.a.	0	0	0.05	0
**VAL-III**	4.03	0.04	0.88	n.a.	0.38	0	0.05	0
**Methanogens**	0.88	2.32	0.52	n.a.	0.47	6.58	0.05	1.28
**DSEG**	0.29	0.14	0.36	n.a.	0.08	0	0	0
**MBG-E**	0	0.29	0.05	n.a.	0.07	0	0	0
**MG-IV**	0	0.11	0	n.a.	0	0.18	0	0.14
**VAL-II**	0.82	3.46	1.42	n.a.	0.41	1.17	0.99	1.01
**RC-III**	1.69	0.39	0.2	n.a.	0	0.46	0	0
**Group_1.1c**	0	0.29	0	n.a.	0	1.13	0	0.1
**Unclassified**	1.74	1.01	0.35	n.a.	0.25	2.16	0.07	2.22
**Total # sequences**	1717	2803	4433	188	7293	2827	2216	7131

	Groundwater samples – June 2014							
	H3-1		H4-1		H5-1		H3-2	
	**RNA** (%)	**DNA** (%)	**RNA** (%)	**DNA** (%)	**RNA** (%)	**DNA** (%)	**RNA** (%)	**DNA** (%)
**FSCG**	0.03	0.04	0	0.29	0.01	0.44	0	0.26
**SCG**	0	0.02	0	0.03	0.13	0	0.13	0.01
**MG-I**	63.29	59.98	44.60	29.08	18.22	14.71	3.41	10.68
**RC-V**	26.67	15.08	31.49	29.02	40.65	39.28	62.11	47.35
**DHVE-6**	5.01	21.78	6.78	26.05	19.34	34.55	16.51	31.37
**Cand. Iainarchaeum**	1.05	0.78	11.78	6.99	3.10	3.81	2.04	2.17
**SITS412**	0.69	0.23	1.07	2.65	1.28	1.16	0.68	0.91
**Hadesarchaea**	0	0.01	0	0	0.05	0.03	0.10	0
**MBG-A**	0.01	0	0.09	0.13	0	0.13	0.06	0.08
**pSL12**	0.05	0.06	0.09	0	0.06	0.06	0.06	0.10
**SAGMCG**	0.61	1.32	1.34	3.07	1.71	2.65	1.49	4.39
**MCG-6**	0	0.01	0	0	0.02	0.07	0	0
**MCG-11**	0.01	0.01	0	0	0.26	0.24	0	0.03
**VAL-III**	0.85	0.03	0.71	0.10	1.15	0.30	1.59	0.06
**Methanogens**	0.24	0.47	0.89	0.39	10.38	1.14	7.91	1.53
**DSEG**	0.03	0.04	0.09	0.20	0.02	0.15	0	0.13
**MBG-E**	0.01	0.03	0.27	0.75	0.01	0.04	0.13	0.09
**VAL-II**	1.37	0.10	0.80	0.52	2.69	0.86	2.47	0.50
**Unclassified**	0.08	0.01	0	0.73	0.92	0.38	1.31	0.34
**Total # sequences**	14333	11863	1121	3060	8190	6752	3083	10001

	Groundwater samples – June 2014							
	**H4-2**		**H4-3**		**H5-2**		**H5-3**	
	**RNA** (%)	**DNA** (%)	**RNA** (%)	**DNA** (%)	**RNA** (%)	**DNA** (%)	**RNA** (%)	**DNA** (%)
**SCG**	0.07	0	0	0.06	0	0.05	0	0
**MG-I**	2.88	1.67	4.01	3.32	24.24	18.93	90.67	87.87
**RC-V**	40.45	27.92	59.36	41.44	68.13	40.85	4.48	5.78
**DHVE-6**	15.63	35.10	21.05	38.74	5.40	25.11	0.84	2.62
**Cand. Iainarchaeum**	28.11	25.76	5.27	3.89	0.14	2.30	0.19	0.09
**SITS412**	2.53	3.61	4.85	7.21	0.14	0.18	1.68	0.18
**Hadesarchaea**	0	0.08	0.05	0.06	0	0.71	0	0.12
**MBG-A**	0	0.13	0	0.73	0	0.03	0	0
**pSL12**	0	0.01	0	0.06	0	0.03	0	0
**SAGMCG**	0.19	0.08	0	0.39	0.27	1.03	0.09	0.48
**MCG-6**	0.19	0.59	0.33	0.34	0	0	0	0
**MCG-11**	0.16	0.78	0.05	0.90	0	0.15	0	0.03
**VAL-III**	4.15	0.40	2.66	0.17	0.47	0.03	0.09	0.03
**Methanogens**	2.18	0.70	0.56	0.73	0.68	9.26	0	0.15
**DSEG**	0.16	0.09	0.33	0.23	0	0.03	0.28	0.03
**MBG-E**	0	0.18	0	0.06	0	0.10	0	0
**VAL-II**	0.79	0.59	0.61	0.51	0.34	0.63	1.59	2.35
**Unclassified**	2.51	2.31	0.87	1.16	0.19	0.58	0.09	0.27
**Total # sequences**	4312	7736	2143	1776	1481	3963	1072	3355

	Groundwater samples – September 2014							
	H3-1		H4-1		H5-1		H3-2	
	**RNA** (%)	**DNA** (%)	**RNA** (%)	**DNA** (%)	**RNA** (%)	**DNA** (%)	**RNA** (%)	**DNA** (%)
**FSCG**	n.a.	n.a.	0.02	0.06	0.03	0.40	0.09	0.12
**SCG**	n.a.	n.a.	0.02	0.03	0.05	0.04	0.36	0.10
**MG-I**	n.a.	n.a.	41.90	25.01	30.72	47.76	2.47	4.66
**RC-V**	n.a.	n.a.	32.80	28.79	46.16	23.57	68.85	43.05
**DHVE-6**	n.a.	n.a.	10.31	25.94	14.47	17.67	8.63	40.26
**Cand. Iainarchaeum**	n.a.	n.a.	6.98	4.50	2.19	2.89	4.09	2.16
**SITS412**	n.a.	n.a.	4.07	6.20	0.88	1.64	2.11	0.99
**Hadesarchaea**	n.a.	n.a.	0	0.03	0.01	0.04	0	0
**MBG-A**	n.a.	n.a.	0.04	0.03	0.03	0.04	0	0
**pSL12**	n.a.	n.a.	0.09	0.46	0.01	0.44	0.09	0.17
**SAGMCG**	n.a.	n.a.	0.72	3.69	1.71	3.28	1.66	2.93
**MCG-6**	n.a.	n.a.	0	0	0.02	0.09	0	0
**MCG-11**	n.a.	n.a.	0	0	0.05	0.36	0	0.07
**VAL-III**	n.a.	n.a.	0.89	0.23	0.90	0.31	8.31	0.12
**Methanogens**	n.a.	n.a.	0.56	2.45	0.81	0.58	0.76	4.29
**DSEG**	n.a.	n.a.	0.04	0	0.11	0.04	0.04	0.05
**MBG-E**	n.a.	n.a.	0.45	0.69	0.01	0	0.09	0.07
**VAL-II**	n.a.	n.a.	0.96	0.78	1.71	0.44	2.34	0.36
**Unclassified**	n.a.	n.a.	0.15	1.11	0.13	0.41	0.11	0.6
**Total # sequences**	n.a.	n.a.	5528	3470	15822	2253	2225	4123

	**Groundwater samples – September 2014**							
	**H4-2**		**H4-3**		**H5-2**		**H5-3**	
	**RNA** (%)	**DNA** (%)	**RNA** (%)	**DNA** (%)	**RNA** (%)	**DNA** (%)	**RNA** (%)	**DNA** (%)
**FSCG**	n.a.	0	0	0	0	0.03	n.a.	0
**SCG**	n.a.	0	0.03	0	0.05	0	n.a.	0.04
**MG-I**	n.a.	1.07	0.87	1.61	96.35	39.91	n.a.	93.44
**RC-V**	n.a.	23.43	58.65	32.02	2.17	32.62	n.a.	2.85
**DHVE-6**	n.a.	26.39	21.95	53.45	0.20	23.47	n.a.	1.92
**Cand. Iainarchaeum**	n.a.	38.54	6.47	4.90	0.05	0.94	n.a.	0.06
**SITS412**	n.a.	4.92	6.87	4.38	0.53	0.26	n.a.	0.22
**Hadesarchaea**	n.a.	0.08	0	0	0	0.06	n.a.	0.02
**MBG-A**	n.a.	0.08	0.03	0.15	0.01	0.03	n.a.	0
**pSL12**	n.a.	0	0	0.06	0	0	n.a.	0
**SAGMCG**	n.a.	0.10	0.10	0.11	0.09	0.91	n.a.	0.38
**MCG-6**	n.a.	0.44	0.27	0.15	0	0	n.a.	0
**MCG-11**	n.a.	1.33	0.05	0.47	0	0.06	n.a.	0
**VAL-III**	n.a.	0.56	1.35	0.18	0.05	0.09	n.a.	0
**Methanogens**	n.a.	0.36	0.83	0.80	0.02	0.88	n.a.	0.12
**DSEG**	n.a.	0.18	0.10	0.44	0.02	0.09	n.a.	0
**MBG-E**	n.a.	0.08	0.18	0.11	0	0	n.a.	0
**VAL-II**	n.a.	0.56	1.22	0.54	0.43	0.35	n.a.	0.89
**Unclassified**	n.a.	1.88	1.03	0.63	0.03	0.3	n.a.	0.06
**Total # sequences**	n.a.	4964	7321	6649	13327	3400	n.a.	8324

Table S3.

Month sampled	Well	Oxygen saturation (%)	NO_3^- (mg/L)	NH_4 (mg/L)
**June 2014**	H3-2	27.1	27.72	0.04
	H4-2	0	0	0.20
	H4-3	0	0	0.12
	H5-2	0	9.56	0.43
	H5-3	0	0	0.75
	H3-1	31.9	28.53	0.01
	H4-1	37.4	14.50	0.11
	H5-1	25.3	12.21	0.10
**August 2014**	H3-2	21.7	40.21	0.08
	H4-2	0	0	0.31
	H4-3	0	0	0.20
	H5-2	0	0	0.47
	H5-3	0.2	0	0.77
	H3-1	43.3	32.69	0.12
	H4-1	43.6	17.06	0.23
	H5-1	29.1	15.46	0.01
**September 2014**	H3-2	17.2	32.33	0
	H4-2	0	0	0.15
	H4-3	0	0	0.05
	H5-2	0	0	0.51
	H5-3	0	0	0.77
	H4-1	51	9.84	0.13
	H5-1	30.3	8.45	0.03

Table S4.

SIP using filtered groundwater from site H4 (wells H4-1 and H4-3)

	**$^{13}CO_2$_Duplicate 1_4 weeks_H41 (B9_4w)**				**$^{13}CO_2$_Duplicate 2_4 weeks_H41 (B10_4w)**				**$^{12}CO_2$_Control_4 weeks_H41 (B11_4w)**		
	RoOR	OR	Heavy Fraction	Light Fraction	RoOR	OR	Heavy Fraction	Light Fraction	OR	Heavy Fraction	Light Fraction
**MG-I (Group 1.1a)_Thaumarchaeota**	0.71	1.14	590	2227	0.81	1.30	297	966	1.61	206	1354
**RC-V_Woesearchaeota**	1.32	0.89	44	192	0.76	0.51	17	111	0.67	19	201
**DHVE-6_Woesearchaeota**	**2.07**	3.44	64	77	**1.3**	2.16	24	40	1.66	8	36
**SAGMCG (Group 1.1a)_Thaumarchaeota**	0.91	1.18	47	156	**2.04**	2.66	36	50	1.31	20	115
**Total # reads**	na	na	764	2974	na	na	387	1347	na	258	1903

	**$^{13}CO_2$_Duplicate 1_4 weeks_H41 (B9_8w)**				**$^{13}CO_2$_Duplicate 2_4 weeks_H41 (B10_8w)**				**$^{12}CO_2$_Control_4 weeks_H41 (B11_8w)**		
	RoOR	OR	Heavy Fraction	Light Fraction	RoOR	OR	Heavy Fraction	Light Fraction	OR	Heavy Fraction	Light Fraction
**MG-I (Group 1.1a)_Thaumarchaeota**	0.06	0.37	1076	4193	0.11	0.69	455	1323	6.16	224	63
**DHVE-6_Woesearchaeota**	**50.19**	18.02	53	11	**22.66**	8.13	25	9	0.36	5	5
**SAGMCG (Group 1.1a)_Thaumarchaeota**	**4.19**	1.52	43	103	**2.76**	1.00	16	45	0.36	5	5
**Total # reads**	na	na	1230	4413	na	na	514	1441	na	239	89

	**$^{13}CO_2$_Duplicate 1_12 weeks_H41 (B9)**				**$^{13}CO_2$_Duplicate 2_12 weeks_H41 (B10)**				**$^{12}CO_2$_Control_12 weeks_H41 (B11)**		
	RoOR	OR	Heavy Fraction	Light Fraction	RoOR	OR	Heavy Fraction	Light Fraction	OR	Heavy Fraction	Light Fraction
**MG-I (Group 1.1a)_Thaumarchaeota**	0.21	0.24	1842	9972	0.14	0.16	427	1827	1.11	845	723
**RC-V_Woesearchaeota**	**5.23**	3.39	425	548	**6.29**	4.08	134	101	0.65	71	92
**DHVE-6_Woesearchaeota**	**6.57**	7.83	493	290	**5.98**	7.13	132	58	1.19	34	25
**SAGMCG (Group 1.1a)_Thaumarchaeota**	0.91	0.99	45	178	**1.38**	1.51	19	34	1.09	35	28
**Total # reads**	na	na	2884	11301	na	na	771	2060	na	1021	890

	**^{13}C-VA_Duplicate 1_12 weeks_H41 (B17)**				**^{13}C-VA_Duplicate 2_12 weeks_H41 (B18)**				**^{12}C-VA_Control_12 weeks_H41 (B19)**		
	RoOR	OR	Heavy Fraction	Light Fraction	RoOR	OR	Heavy Fraction	Light Fraction	OR	Heavy Fraction	Light Fraction
**MG-I (Group 1.1a)_Thaumarchaeota**	na	na	na	na	**6.26**	3.94	310	325	0.63	103	425
**RC-V_Woesearchaeota**	na	na	na	na	0.78	0.42	20	58	0.54	6	39
**Total # reads**	na	na	na	na	na	na	340	449	na	148	542

	$^{13}CO_2$_Duplicate 1_4 weeks_H43 (B1_4w)				$^{13}CO_2$_Duplicate 2_4 weeks_H43 (B2_4w)				$^{12}CO_2$_Control_4 weeks_H43 (B3_4w)		
	RoOR	OR	Heavy Fraction	Light Fraction	RoOR	OR	Heavy Fraction	Light Fraction	OR	Heavy Fraction	Light Fraction
**MG-I (Group 1.1a)_Thaumarchaeota**	0.77	1.79	170	138	0.98	2.3	187	368	2.34	697	596
**RC-V_Woesearchaeota**	**1.87**	1.43	436	468	**1.43**	1.09	409	2002	0.76	862	1831
**SITS412_Euryarchaeota**	0.90	0.13	84	610	1.41	0.20	197	3194	0.14	226	1992
**DHVE-6_Woesearchaeota**	0.68	2.62	406	269	1.70	6.54	366	384	3.84	1068	646
**Methanogens_Euryarchaeota**	0.40	0.53	7	18	2.00	2.61	13	26	1.31	29	39
**Subgroup 11 (MCG-11)_Bathyarchaeota**					0.90	1.05	18	89	1.17	24	36
**Subgroup 6 (MCG-6)_Bathyarchaeota**	**8.09**	1.48	13	12	**5.89**	1.08	11	53	0.18	6	57
**Total # reads**	na	na	1173	1599	na	na	1277	6632	na	3224	5659

	$^{13}CO_2$_Duplicate 1_8 weeks_H43 (B1_8w)				$^{13}CO_2$_Duplicate 2_8 weeks_H43 (B2_8w)				$^{12}CO_2$_Control_8 weeks_H43 (B3_8w)		
	RoOR	OR	Heavy Fraction	Light Fraction	RoOR	OR	Heavy Fraction	Light Fraction	OR	Heavy Fraction	Light Fraction
**MG-I (Group 1.1a)_Thaumarchaeota**	0.06	1.10	164	310	0.15	2.87	669	378	18.95	277	96
**RC-V_Woesearchaeota**	0.07	0.83	322	726	0.14	1.74	947	869	12.66	389	227
**SITS412_Euryarchaeota**	0.07	0.07	11	280	0.09	0.08	246	2176	0.90	62	343
**DHVE-6_Woesearchaeota**	0.23	6.01	202	89	0.19	5.13	800	276	26.44	309	80
**MCG subgroups_Bathyarchaeota**	0.37	0.48	5	21	0.58	0.76	35	63	1.32	6	23
**Total # reads**	na	na	738	1508	na	na	2927	4037	na	1124	812

	$^{13}CO_2$_Duplicate 1_12 weeks_H43 (B1)				$^{13}CO_2$_Duplicate 2_12 weeks_H43 (B2)				$^{12}CO_2$_Control_12 weeks_H43 (B3)		
	RoOR	OR	Heavy Fraction	Light Fraction	RoOR	OR	Heavy Fraction	Light Fraction	OR	Heavy Fraction	Light Fraction
**MG-I (Group 1.1a)_Thaumarchaeota**	na	na	na	na	0.94	1.37	252	749	1.46	48	15
**RC-V_Woesearchaeota**	na	na	na	na	**1.04**	0.66	519	2685	0.63	168	100
**SITS412_Euryarchaeota**	na	na	na	na	2.65	0.30	159	1739	0.11	17	54
**DHVE-6_Woesearchaeota**	na	na	na	na	0.79	4.41	713	909	5.59	270	36
**Cand. Iainarchaeum_Diapherotrites**	na	na	na	na	1.84	1.56	30	76	0.85	25	13
**Total # reads**	na	na	na	na	na	na	1824	7161	na	549	244

SIP using passive sampler material exposed in well H4-1

	$^{13}CO_2$_Duplicate 1 (B2p)				$^{13}CO_2$_Duplicate 2 (B6p)				$^{12}CO_2$_Control (B12p)		
	RoOR	OR	Heavy Fraction	Light Fraction	RoOR	OR	Heavy Fraction	Light Fraction	OR	Heavy Fraction	Light Fraction
**SITS412_Euryarchaeota**	na	na	na	na	1.68	0.09	1354	4595	0.05	144	210
**MG-I (Group 1.1a)_Thaumarchaeota**	na	na	na	na	**5.06**	2.60	20	23	0.51	54	67
**SCG (Group 1.1b)_Thaumarchaeota**	na	na	na	na	**87.46**	17.74	162	30	0.20	5	14
**Total # reads**	na	na	na	na	na	na	1572	4662	na	297	222

SIP using filtered groundwater from site H5 (wells H5-1 and H5-2)

	**$^{13}CO2$_Duplicate 1_5 weeks_oxic (B1_H5)**				**$^{13}CO2$_Duplicate 2_12 weeks_oxic (B2_H5)**				**$^{12}CO2$_Control_12 weeks_oxic (B3_H5)**		
	RoOR	OR	Heavy Fraction	Light Fraction	RoOR	OR	Heavy Fraction	Light Fraction	OR	Heavy Fraction	Light Fraction
**MG-I (Group 1.1a)_Thaumarchaeota**	0.03	0.16	24264	53577	0.03	0.17	31738	44817	5.5	28165	12779
**Unclassified Thaumarchaeota**	**1858.15**	4.39	163	81	**2148.11**	5.07	971	258	0.0024	6	1297
**SAGMCG (Group 1.1a)_Thaumarchaeota**	**6899.15**	8.20	158	42	**3738.53**	4.45	258	286	0.0012	5	2040
**Total # reads**	na	na	24791	53763	na	na	34537	45483	na	29619	16408

	**$^{13}CO2$_Duplicate 1_5 weeks_anoxic (B11_H5)**				**$^{13}CO2$_Duplicate 2_5 weeks_anoxic (B12_H5)**				**$^{12}CO2$_Control_5 weeks_anoxic (B13_H5)**		
	RoOR	OR	Heavy Fraction	Light Fraction	RoOR	OR	Heavy Fraction	Light Fraction	OR	Heavy Fraction	Light Fraction
**MG-I (Group 1.1a)_Thaumarchaeota**	0.01	0.04	26136	27132	0.23	1.16	16703	19562	5.06	29793	50545
**Unclassified Thaumarchaeota**	**943.96**	443.93	2766	6	**1.93**	0.91	422	548	0.47	8	29
**Total # reads**	na	na	31131	27321	na	na	17898	21179	na	29832	50871

Table S5.

				DOC (mg/L)		DIC (mg/L)		O_2 (mg/L)		NH_4^+ (mg/L)		NO_3^- (mg/L)		SO_4^{2-} (mg/L)	
Well	Substrate		Sample	T0	Te	T0	Te	T0	Te	T0	Te	T0	Te	T0	Te
H4-3	13CO2_1	Anoxic	B1	4.3	2.5	na	na	na	na	0.1	nd	0.4	<0.1	39.3	39.2
H4-3	13CO2_2	Anoxic	B2	4.3	2.7	na	na	na	na	0.1	nd	0.4	nd	39.3	37.8
H4-3	12CO2	Anoxic	B3	4.3	2.3	na	na	na	na	0.1	nd	0.4	<0.1	39.3	41.1
H5-2	13CO2_1	Anoxic	B1_H5	0.7	0.9	na	na	na	na	0.4	nd	3.1	1.3	95.3	89.7
H5-2	13CO2_2	Anoxic	B2_H5	0.7	1.8	na	na	na	na	0.4	<0.1	3.1	1.8	95.3	86.4
H5-2	12CO2	Anoxic	B3_H5	0.7	1.3	na	na	na	na	0.4	<0.1	3.1	0.7	95.3	89
H4-1	13CO2_1	Oxic	B9	1.4	2.2	na	na	8.6	7.9	<0.1	<0.1	11.6	13.4	104.7	111.6
H4-1	13CO2_2	Oxic	B10	1.4	2.5	na	na	8.6	7.8	<0.1	<0.1	11.6	13.7	104.7	115.2
H4-1	12CO2	Oxic	B11	1.4	2.2	na	na	8.2	6.8	<0.1	<0.1	11.6	12.9	104.7	109.6
H4-1	13C-VA_1	Oxic	B17	na	na	72.2	65.4	8.9	7.9	<0.1	<0.1	11.6	11.8	104.7	110.5
H4-1	13C-VA_2	Oxic	B18	na	na	72.2	66.4	8.8	7.8	<0.1	<0.1	11.6	10.9	104.7	107.7
H4-1	12C-VA	Oxic	B19	na	na	72.2	50.2	8.8	7.8	<0.1	<0.1	11.6	10.3	104.7	107.2
H5-1	13CO2_1	Oxic	B11_H5	0.9	1.4	na	na	8.	7.4	<0.1	<0.1	7	6.8	316.4	296.1
H5-1	13CO2_2	Oxic	B12_H5	0.9	1.6	na	na	8.2	7.4	<0.1	<0.1	7	7	316.4	303.1
H5-1	12CO2	Oxic	B13_H5	0.9	1.8	na	na	8.2	8	<0.1	<0.1	7	6.9	316.4	297.8

Table S6.
